# A novel method for effectively selecting fragments not associated with restriction sites for whole-genome genotyping

**DOI:** 10.1186/s12915-025-02330-8

**Published:** 2025-10-30

**Authors:** Peng Chen, Bipei Zhang, Sheng Zhao, Zhenghang Zhu, Yiming Yan, Haotian Chen, Hong Lu, Yong Xiang, Yongquan Li, Yuxiao Chang

**Affiliations:** 1 Guangdong Laboratory of Lingnan Modern Agriculture, Genome Analysis Laboratory of the Ministry of Agriculture and Rural Affairs Agricultural Genomics Institute at Shenzhen, Chinese Academy of Agricultural Sciences, Shenzhen, 518120 China; 2 College of Horticulture and Landscape Architecture, Zhongkai University of Agriculture and Engineering, Guangzhou, 510225 China; 3 College of Agronomy, Anhui Agricultural University, Hefei, 230036 China

**Keywords:** Reduced representation sequencing, Whole-genome genotyping, Molecular breeding, Restriction site-associated DNA sequencing, Tn5

## Abstract

**Background:**

Reduced representation sequencing (RRS) using Restriction site-associated DNA sequencing (RAD-seq) has become a widely adopted method for whole-genome genotyping, owing to its cost-effectiveness and applicability across species. However, traditional RAD-seq approaches face challenges, including complex workflows and high labor demands. Existing RAD-seq methods rely primarily on the strategy of “selecting fragments first, then preparing a library,” which involves targeting fragments associated with restriction enzyme cut sites.

**Results:**

In contrast, we developed inverse RAD-seq (iRAD-seq), a novel method that employs a “prepare library first, then select” strategy to efficiently capture fragments not associated with restriction sites for whole-genome genotyping. This innovative approach utilizes Tn5 transposase to simultaneously fragment DNA and ligate adapters, followed by pooled processing of hundreds of libraries for batch restriction digestion, thereby significantly streamlining RAD-seq library preparation. This simplified workflow makes iRAD-seq highly compatible with liquid handling automation, enhancing its throughput. We validated iRAD-seq through both in silico analyses and wet-lab experiments in maize and rice. The results demonstrate that iRAD-seq provides consistent genome-wide Single-Nucleotide Polymorphism (SNP) distributions. In maize germplasm, iRAD-seq successfully enables effective genetic diversity analysis. Furthermore, genetic mapping of maize populations has confirmed its utility for identifying quantitative trait loci (QTL).

**Conclusions:**

Our findings suggest that iRAD-seq offers a more streamlined, efficient, and flexible approach to genotyping and marker development than traditional RAD-seq methods. Owing to its potential for high-throughput applications and cost reductions, iRAD-seq is a valuable tool for genomic research and molecular breeding.

**Supplementary Information:**

The online version contains supplementary material available at 10.1186/s12915-025-02330-8.

## Background

Genome-wide single nucleotide polymorphism (SNP) discovery and genotyping are essential for breeding research [1–3]. However, for many projects, genotyping via whole genome sequencing is expensive and generates more SNPs than needed. Therefore, the strategy of reduced representation sequencing (RRS), which selects only the partial genome (usually 1–10%) for sequencing [4–6], is more popular for genome-wide molecular marker detection in breeding projects.

Restriction site-associated DNA sequencing (RAD-seq) is a classical RRS method. In the original RAD-seq protocol, genomic DNA is digested with a RE and ligated with the P1 adapter containing a barcode; then, the barcoded fragments are pooled, randomly sheared, and size-selected; after that, the P2 adapters are ligated to the pooled fragments. Finally, the fragments with both P1 and P2 adapters are amplified and sequenced [7]. Although this RAD-seq method has proven to be an effective method for selecting partial genomes and has been used for genetics and breeding research in various species, its library preparation process has been too tedious, and efforts to improve it never stopped [7, 8].

For example, double digest-RAD (dd-RAD) elaborates the original RAD-seq by eliminating random shearing and explicitly using size selection, hence only regions adjacent to restriction enzyme (RE) recognition sites are chosen for sequencing [9]. More than 90% of REs belong to the type IIP subtype, which recognizes and cleaves symmetric (or “palindromic”) DNA sequences 4 to 8 base pairs in length. In contrast, Type IIB REs cleave DNA upstream and downstream ~ 7–15 bp away from their specific recognition site and generate ~ 30 bp DNA fragments with recognition sites in the central region [10]. 2b-RAD uses the type IIB restriction endonucleases to digest genomic DNA and recover fragments of uniform length for library preparation and sequencing [11]. Similar improvements in RAD-seq also include ezRAD [12]. However, the original RAD-seq method and its related derivatives are still based on a tedious multistep separation and purification process, which usually takes a long time and has high labor costs when dealing with large cohorts of samples. In addition, the ezRAD and ddRAD techniques incorporated size selection after restriction enzyme digestion. As designed, these methods exclusively perform size selection at the single-sample level, which may introduce inter-sample variability in fragment recovery efficiency, potentially leading to a significant loss of markers. As this selection is random, experimental repeatability is poor, making it difficult to control the number of makers [8]. Therefore, fast, cost-effective and high-throughput genotyping techniques are needed.

The RRS strategy contains two essential steps, as demonstrated in traditional RAD-seq and its derivatives: one is how to “select” the fractional representative DNA fragments, and the other is how to add adapters to the fragments for sequencing. Although these RAD-seq methods differ in the type of REs and ligated adapters used, they all prioritize selecting regions adjacent to the RE recognition site before preparing the sequence library. The step of adapter ligation by T4 ligase during the library preparation process is time-consuming, labor-intensive, and inefficient. As a result, RAD-seq methods also suffer from these drawbacks.

An alternative method for adapter ligation that is simple and highly efficient involves the use of the Tn5 transposase, which randomly cleaves genomic DNA and ligates adapters [13–15]. Previously, we reported the All-in-one sequencing (AIO-seq), an improved library preparation method with Tn5. With this method, hundreds of samples can be pooled after PCR for size selection and quantification [16]. On this basis, we propose a novel RRS strategy called Inverse Restriction site Associated DNA sequencing (iRAD-seq). This represents an improvement over current RAD-seq methods. Unlike the conventional RAD-seq strategy, which selects fragments first and then prepares the library, our iRAD-seq follows an inverse approach: preparing the library first and then selecting the fragments. The introduction of Tn5 enables one-step fragmentation and adapter addition, simplifying the library construction process and saving time [13–15]. Our iRAD-seq method, which is based on this strategy and combined with our AIO-seq method, offers a more effective and easier method for RRS library preparation.

## Results

### The iRAD-seq workflow

**Fig. 1:**
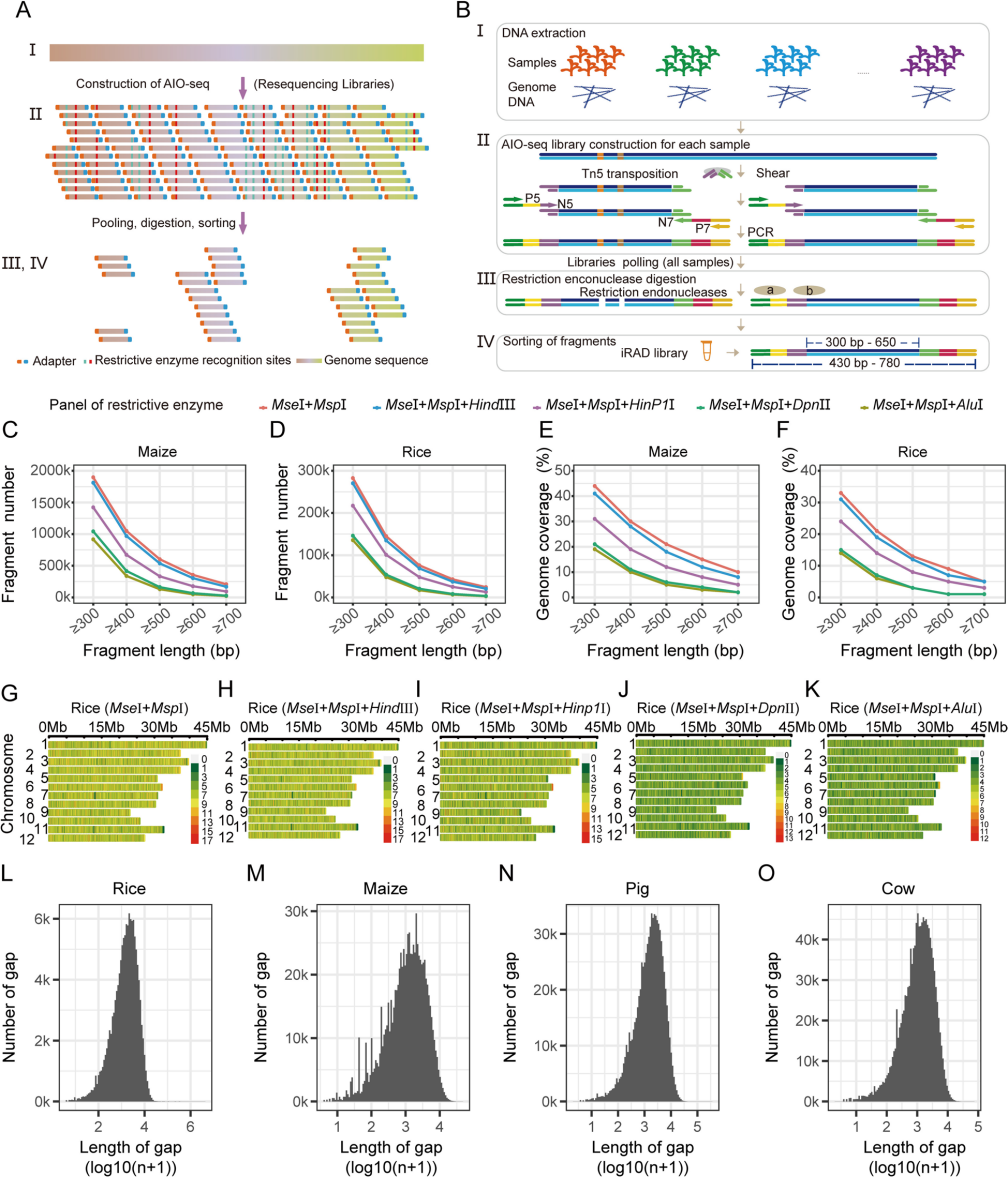
Overview and in silico digestion evaluation of iRAD-seq. **A** Schematic of the iRAD-seq method. After preparation of the AIO-seq library (I), the fragments were digested using REs, and the digestion products were sorted (II) and then sequenced on the next-generation sequencing platform (III, IV). The colors in I, II, and III are in a corresponding relationship. **B** The detailed experimental process of iRAD-seq. Extraction of DNA from samples (I). Genomic DNA was digested with Tn5 transposase and the sequencing adapter was added to the fragments by PCR (II). Fragments were digested with 2–3 REs, and the digested fragments could not be sequenced (III). The digestion products were sorted and then sequenced on the next-generation sequencing platform (IV). Steps I-IV represent identical experimental workflow in both A and B. **C**-**D** The number of fragments of different lengths in maize and rice. **E**–**F** Genome coverage analysis of fragments of different lengths in maize and rice. **G**-**K** Chromosome distribution of fragments (length > 300 bp). The rice genome was digested by different panels of REs. The size of sliding window was 10 kb. Red, high density; green, low density. **L**-**O** Histogram of the number of gaps in rice, maize, pig (susScr11), and cow (bosTau9). *Mse*I, *Msp*I, and *Alu*I were selected for in silico digestion of the genome. The gap length in the genome is defined as the distance between fragments (length > 300 bp)

Our goal was to develop a novel RAD-seq method capable of identifying genome-wide SNP markers, utilizing the AIO-seq method [16] and RE reduced representation sequencing. Our iRAD-seq protocol involves the following steps: initially, a whole-genome resequencing library with a unique dual-index was prepared for each sample using Tn5 transposase, following the AIO-seq protocol; Subsequently, the dual-indexed libraries were pooled and subjected to enzymatic digestion with a panel of REs; Fragments containing the respective restriction sites were cleaved into smaller pieces and filtered by size selection. Following digestion, fragments ranging from approximately 430 bp to 780 bp (including the insertion and 136 bp adaptors) were size-selected for sequencing (Fig. 1A and B). Despite some fragments fell within this length range after enzyme cleavage, they could still not be sequenced due to the absence of the necessary P5 and P7 adapters required for sequencing. By employing this design, genomic fragments devoid of the utilized REs'recognized sites, with lengths ranging from approximately 300 bp to 650 bp, were selected and sequenced to represent the genotyping of the entire genome (Fig. 1B).

### The selection of panel of REs and fragment lengths impacts the efficient of reduced representation

The degree of genome representation (OR simplification) of iRAD-seq can be estimated based on in silico digestion of the reference genome. We selected six REs (*Mse*I, *Msp*I, *Alu*I, *Dpn*II, *Hind*III, and *HinP1*I) to form five panels (*Mse*I + *Msp*I, *Mse*I + *Msp*I + *Alu*I, *Mse*I + *Msp*I + *Dpn*II, *Mse*I + *Msp*I + *Hind*III, and *Mse*I + *Msp*I + *HinP1*I), which underwent in silico digestions of various species including maize (*Zea mays* L.), rice (*Oryza sativa* L.), soybean (*Glycine max* L.), wheat (*Triticum aestivum* L.), pig (*Sus scrofa*) and cow (*Bos taurus*) (Additional file 8: Table S1).

We anticipate that the iRAD-seq method, which utilizes restriction enzymes recognizing 4–6 nucleotide sequences, will be employed. Following this, DNA fragments sized 300–650 bp will be selected through sorting. Among these, fragments containing intact paired-end sequencing adapters (uncut by the enzyme) will be sequenced. These fragments are suitable for paired-end 150 bp sequencing, covering around 10%−20% of the genome, thus can reduce the genomic data by 80%−90%, significantly streamlining the sequencing process and increasing the efficiency of downstream functional genomics and breeding research.

As anticipated, different panels of REs can yield varying degrees of representation (Fig. 1C-F and Additional file 1: Fig. S1A). Specifically, for rice, digestion by the panels of *Mse*I (recognizing TTAA) and *Msp*I (CCGG) produced 282,747 and 145,146 fragments longer than 300 and 400 bp, respectively (Fig. 1D), corresponding to 33.1% and 21.0% genome coverage (Fig. 1F). In addition to *Mse*I and *Msp*I, the inclusion of *Hind*III (AAGCTT) decreased the number of fragments longer than 300 and 400 bp by only 4.3% and 7.0%, respectively (Fig. 1D), resulting in corresponding decreases in genome coverage of 2.0% and 1.8%, respectively (Fig. 1F). However, the addition of *HinP1*I (GCGC), *Dpn*II (GATC), and *Alu*I (AGCT) significantly affected genome reduction, particularly *Dpn*II and *Alu*I, which reduced the final genome coverage to 15.0% and 13.8%, respectively (Fig. 1F). The patterns of soybean, maize, and wheat were similar (Fig. 1C, E and Additional file 1: Figs. S2A-S2D). These findings suggest that our iRAD-seq method is effective, and that various panels of REs can generate different degrees of genome simplification that can meet diverse requirements for marker density.

We then evaluated the uniform distribution of the selected restriction fragments across the genome via in silico digestion. We first calculated the rice genomic fragment distribution selected by different panels of REs across the genome in a 10-kb sliding window and found that the distribution of selected restriction fragments throughout the genome was widespread (Fig. 1G-K and Additional file 1: Fig. S3). We subsequently calculated the gap length between two fragments (> 300 bp) for the genomes of rice, maize, pig and cow digested by *Mse*I, *Msp*I and *Alu*I. The results revealed that gaps were mainly concentrated in regions below 50 kb (Fig. 1L-O); for rice, 22 gaps longer than 50 kb were detected, and the total length of the gaps was 7,952,540 kb, which occupied 2.0% of the total genome (Fig. 1L). Compared with those in rice, the number of gaps longer than 50 kb and the total length of the gaps were lower in maize, pig and cow (Fig. 1M-O). To some extent, these findings reveal that the distribution of fragments in the genome is relatively widespread and that iRAD-seq can meet the requirements of genotype imputation analysis. Taken together, our novel strategy of selecting fragments not associated with restriction sites can be used as an alternative for reduced representation.

### Proof‑of‑concept of iRAD-seq demonstrated with maize and rice

**Fig. 2:**
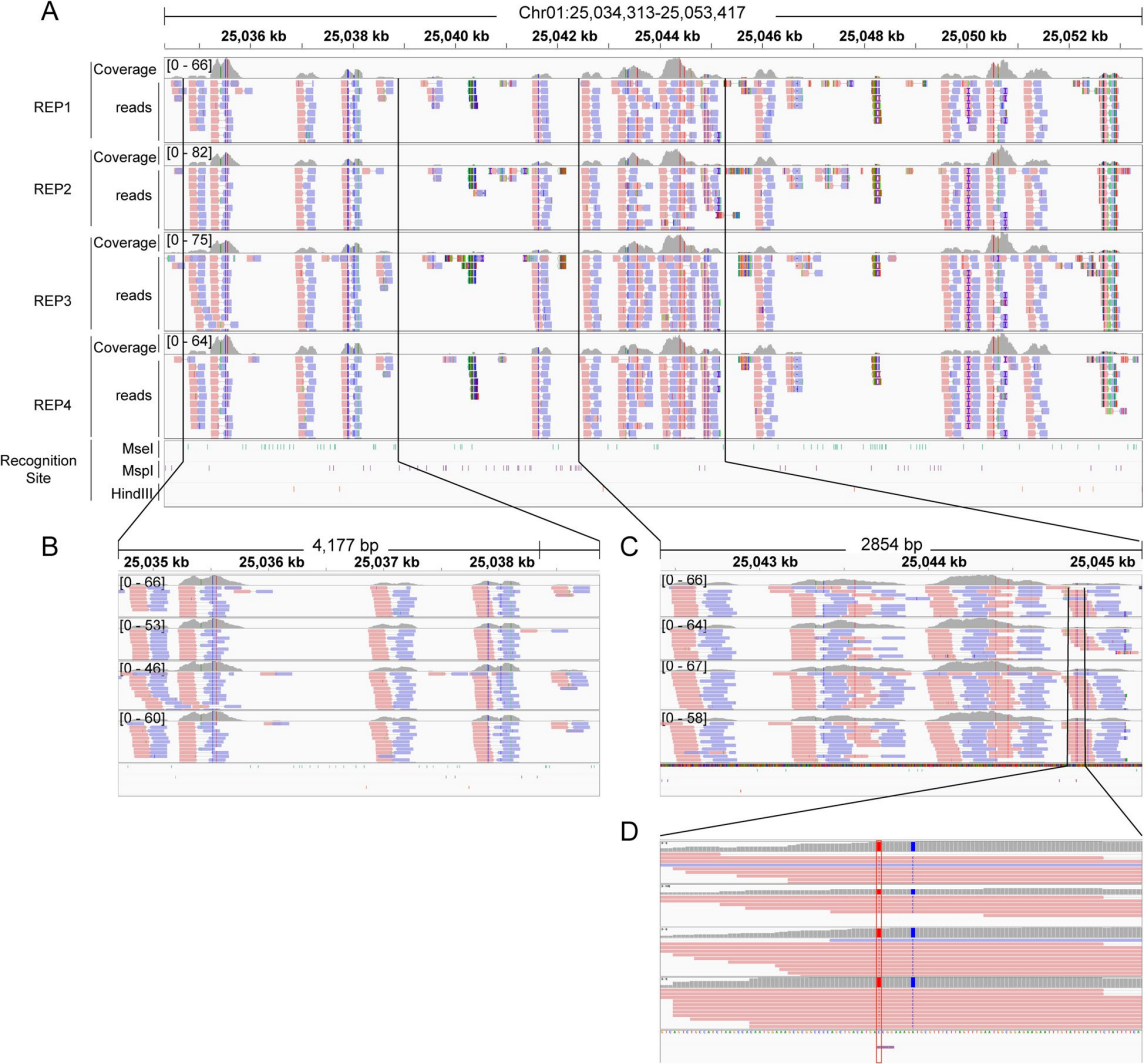
Relationship between sequencing reads and REs recognition sites in the genome. **A**-**C** The reads of iRAD-seq are primarily enriched in non-REs recognition regions (> 300 bp). **D** Reads with mutations in RE recognition sites are enriched in non-RE recognition regions (> 300 bp). The data from rice by iRAD-seq with *Mse*I, *Msp*I and *Hind*III. The black vertical lines indicate the amplified regions. Red boxes indicate bases with mutated of REs recognition sites in reads

To test the feasibility of our iRAD-seq method in a wet lab, we used rice (Nip) and maize (B73) as tests. We initially prepared the AIO-seq library, which was subsequently subjected to RE digestion using two distinct panels: Panel_A (*Alu*I, *Mse*I, *Msp*I) for ​10%−20% genome reduction and Panel_B (*Hind*III, *Mse*I, *Msp*I) for ​30%−50% genome reduction (Fig. 1E-F). Then, we selected fragments range from 430 to 780 bp for sequencing (Fig. 1B). Four technical replicates were carried out for each panel of REs. Sequencing data of 4.2–4.8 Gb were obtained for the maize library, and 2.5–3.0 Gb for the rice library (Additional file 8: Table S2). For the clean data, we first calculated the ratio of reads that still contained the REs’ recognition sites used and found that approximately 0.8%−7.0% of the reads contained these recognition sites. Four technical replicates showed similar result, indicated it maybe the intrinsic property of the REs (Additional file 2: Figs. S4A-S4H). Then, we mapped the reads to the reference and determined the read distribution via the IGV genome browser. As expected, the majority of the reads were located in the region without the REs recognition site (Fig. 2A-C); occasionally, we could find high-depth reads in the region containing the recognition site, but further analysis revealed that SNPs occurred in the test variety compared with the reference, thus losing this recognition site (Fig. 2D). These results showed that the iRAD-seq method effectively reduces genome representation.

**Fig. 3:**
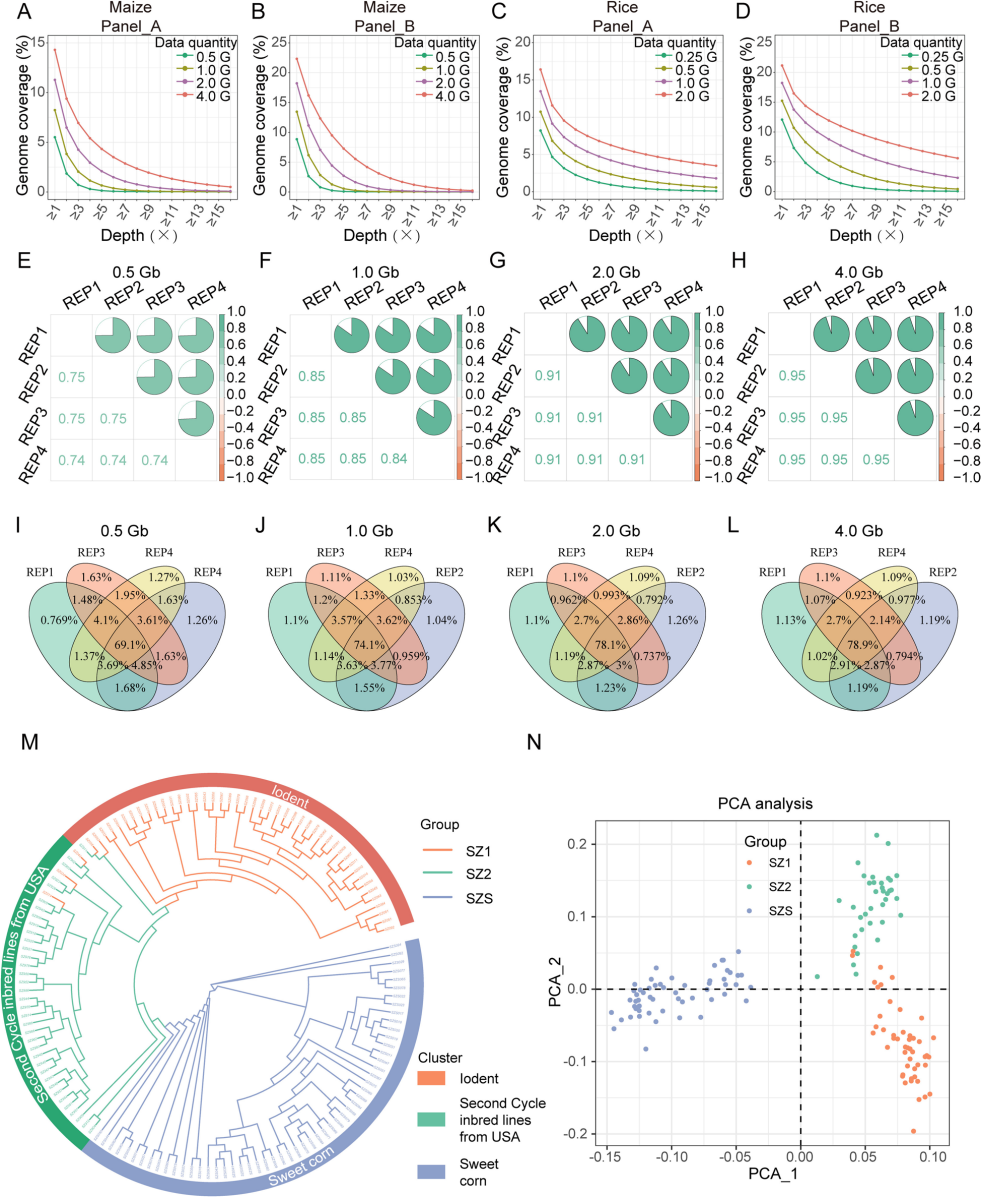
Experiment of iRAD-seq and its utility for maize population structure analysis. **A**-**D** Genome coverage of maize and rice by varying sequencing data sizes. Panel_A (*Alu*I, *Mse*I and *Msp*I) and Panel_B (*Hind*III, *Mse*I and *Msp*I) were selected for iRAD-seq. **E**–**H** Consistency of coverage among 4 repeats when using varying sequencing data sizes. Panel_A was used for iRAD-seq. Pairwise Spearman correlation of the mean coverage between replicates with 100 kb windows. **I**-**L** Number of shared SNPs among 4 repeats when using varying sequencing data sizes. Panel_A was used for iRAD-seq. **M** Neighbor-joining tree of the 139 maize lines based on high-quality SNPs. **N** Principal component analysis of 139 maize lines

To assess the coverage and reproducibility of the target genomic regions of iRAD-seq, we removed reads containing recognition sites of REs and fragments shorter than 300 bp (noise background). We counted the genome coverage using different amounts of data. The wet-lab sequencing data of iRAD-seq exhibit the coverage that is comparable to the results of in silico digestion (Figs. 1E-F and 3A-D). For the data from Panel_A in maize, with 2.0 Gb of sequence data, the genome coverage was 11.3%, increasing the sequencing data to 4.0 Gb and increasing the genome coverage to only 14.3%, which was comparable to in silico digestion prediction of coverage of 18.8% (Figs. 1E and 3A). The digestion by Panel_B in maize and rice followed a similar pattern (Figs. 1E-F, 3B and D). After digestion by Panel_A in rice, the actual genomic coverage at a data volume of 4.0 Gb was 16%, whereas the result of in silico digestion was 14% (Figs. 1F and 3C). We speculate that this discrepancy may be due to mutations occurring at the recognition sites of REs within the sample (Fig. 2D). In summary, the wet-lab data confirmed that the iRAD-seq method effectively selects fragments devoid of recognition sites of REs for genome reduced representation sequencing. Moreover, in silico digestion can be used to guide the selection of RE panels for iRAD-seq.

Finally, we evaluated the reproducibility among the four technical replicates. For maize (2.0 Gb) and rice (2.5 Mb), the Spearman correlation coefficients ranged from 0.88 to 0.95 (Fig. 3E-3 and Additional file 2: Fig. S5). Additionally, SNP overlap across the replicates was 78.1% (Panel A) and 77.6% (Panel B) for maize, and 64.7% (Panel A) and 69.1% (Panel B) for rice. The SNP overlap was positively correlated with the data volume (Fig. 3I-L and Additional file 2: Fig. S6). Collectively, these results highlight the consistent stability and high reproducibility of the iRAD-seq method.

### The application of iRAD-seq in maize germplasm for phylogenetic analysis

In plant breeding, assessing the genetic background of germplasms helps understanding genetic diversity and population structure, providing guidance for subsequent hybridization and selection strategies. To test the performance of iRAD-seq in plant germplasm phylogenetic analysis, we investigated the population genetic structure of 139 maize germplasms by iRAD-seq with Panel_A (*Alu*I, *Mse*I, *Msp*I). We obtained 106.3 Gb (0.6 ± 0.2 Gb per line) of clean data (Additional file 2: Fig. S7A; Additional file 8: Table S3). From this dataset, we identified a total of 1812 high-quality SNPs with a missing rate of less than 20% and a minor allele frequency (MAF) greater than 0.05 (Additional file 8: Table S4). These SNPs are widespread in the genome (Additional file 2: Fig. S7B). An unroot neighbor joining tree showed that the 139 samples were divided into three clusters: “Iodent” (46 lines), “Second Cycle inbred lines from USA” (38 lines) and “Sweet corn” (55 lines) (Fig. 3M). Moreover, we performed principal component analysis (PCA) base on SNP. The samples were also divided into three clusters, exhibiting a clustering pattern identical to the three main clusters found in the phylogenetic analysis. (Fig. 3M-N). Overall, the clear classification of 139 maize lines revealed the ability of iRAD-seq to be used in germplasm genetic background investigations.

### The application of iRAD-seq in maize genetics research

To test the ability of iRAD-seq for genetics research, we perform iRAD-seq with Panel_A and Panel_B for the maize BC_1_F_4_ population of CIMBL83/GEMS41 and the BC_2_F_4_ population of CML496/GEMS41. Since the previous results have showed that approximately 4.6%−7.0% of the reads still contained the RE recognition sites (Additional file 2: Figs. S4A and S4B), thus we performed two rounds of digestion for the Panel_A. For each line, we obtained approximately 2.2 ± 0.8 Gb (Panel_A) and 2.3 ± 0.8 Gb (Panel_B) of sequencing data (Additional file 3: Figs. S8A, S8B; Additional file 8: Table S5). We also sequenced the two populations with low coverage whole genome resequencing by AIO-seq and obtained approximately 2.8 ± 1.2 Gb of sequencing data for each line (Additional file 3: Figs. S8A, S8B; Additional file 8: Table S5). We also sequenced the three parents (CIMBL83, CML496, and GEMS41) and obtained 33.2 Gb, 24.1 Gb and 26.3 Gb raw bases respectively.

**Fig. 4:**
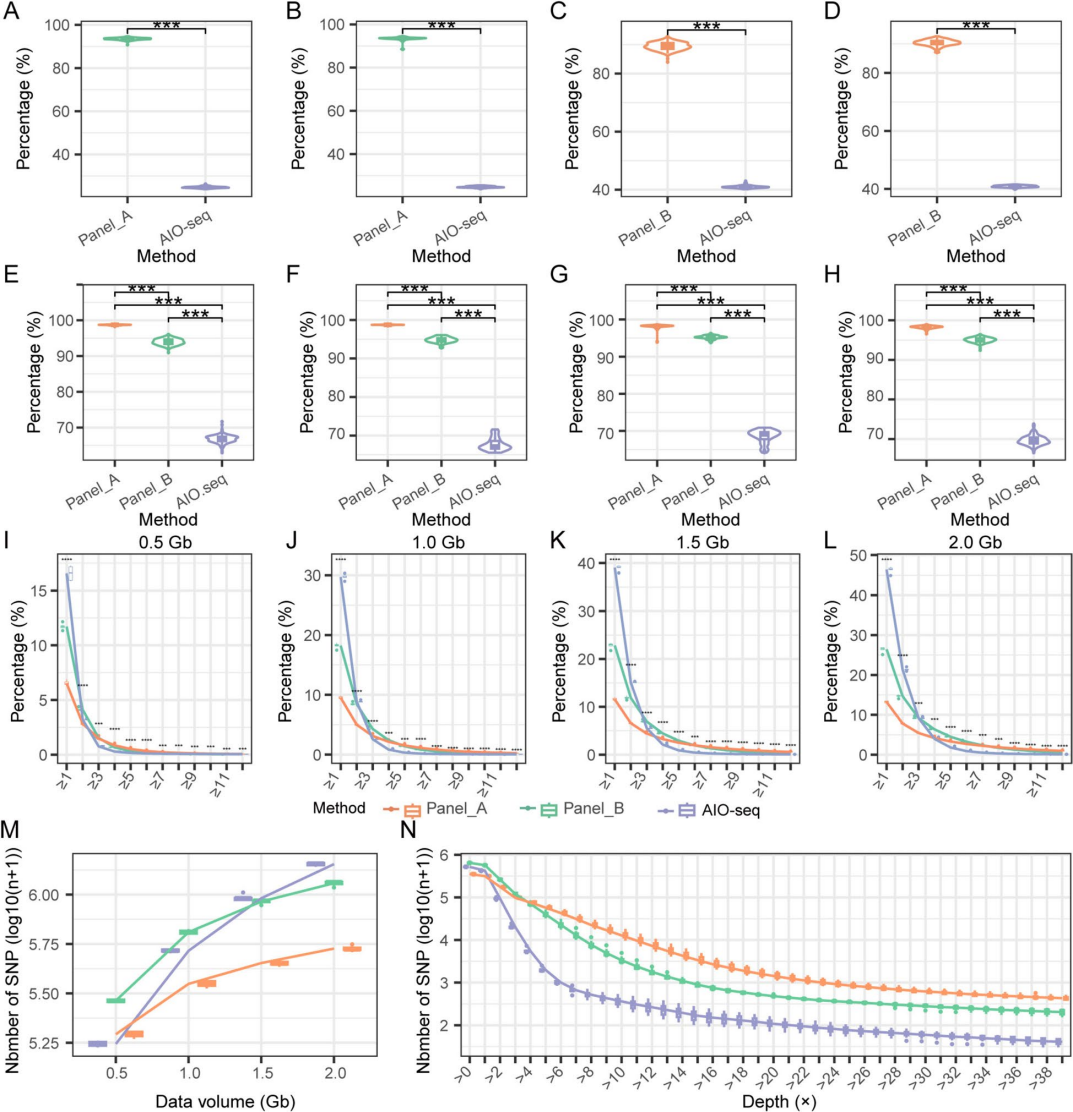
Efficiency of restriction endonucleases and simplified efficiency of iRAD-seq. **A**-**B** Proportion of reads without RE recognition sites in the CIMBL83/GEMS41 population (**A**) and CML496/GEMS41 population (**B**) with Panel_A. **C**-**D** Proportion of reads without RE recognition sites in the CIMBL83/GEMS41 population (**C**) and CML496/GEMS41 population (**D**) with Panel_B. **E**–**F** Proportion of reads without *Mse*I recognition sites in the CIMBL83/GEMS41 population (**E**) and CML496/GEMS41 population (**F**) with Panel_A, Panel_B and AIO-seq. **G**-**H** Proportion of reads without *Msp*I recognition sites in the CIMBL83/GEMS41 population (**G**) and CML496/GEMS41 population (**H**) with Panel_A, Panel_B and AIO-seq. **I**-**L** Genome coverage of iRAD-seq data when using varying sequencing data sizes. **M** The number of SNPs using varying sequencing data sizes. **N** SNP number at different depths. Log10(n + 1): n is the number of SNPs. For boxplots, the center line is the median; box limits represent upper and lower quartiles. Employ the t-test to assess differences in different method, *p* < 0.0001. Different methods use different REs (Panel_A: *Mse*I, *Msp*I and *Alu*I; Panel_B: *Mse*I, *Msp*I and *Hind*III; AIO-seq: none)

For these data, we first analyzed the RE digestion efficiency. On average, across all lines of CIMBL83/GEMS41 and CML496/GEMS41, 93.5% ± 0.7% of the reads in Panel_A and 24.8% ± 0.4% of the reads in AIO-seq did not contain the recognition sites of the REs of Panel_A (Figs. 4A and B). Similarly, 89.6% ± 1.4% of the reads in Panel_B and 40.9% ± 0.4% of the reads in AIO-seq did not contain the recognition sites of the REs of Panel_B (Fig. 4C and D). Panel_A and Panel_B showed reductions of 68.7% ± 0.6% and 48.7% ± 1.4%, respectively, in the number of reads containing recognition sites of REs. Furthermore, we analyzed the digestion efficiency of individual REs. CIMBL83/GEMS41, with averages of 98.7% ± 0.09%, 94.2% ± 0.9%, and 67.0% ± 1.3% of the reads in Panel_A, Panel_B, and AIO-seq, respectively, did not contain MseI recognition sites (Fig. 4E). A similar pattern was observed for *Msp*I, *Alu*I, and *Hind*III (Fig. 4F and Additional file 3: Figs. S9A-S9D). These results showed that iRAD-seq had the remarkable ability to significantly increase the proportion of reads that did not contain recognition sites (*P* < 0.001). Furthermore, the percentage of target reads (without recognition sites of REs) from Panel_A was significantly greater than that from Panel_B (*P* < 0.001, Fig. 4E-H), indicating that the addition of the second round of RE digestion was effective.

To evaluate the efficiency of reduced representation using iRAD-seq, we randomly extracted 0.5, 1.0, 1.5, and 2.0 Gb from the datasets across three strategies in eight individuals. We then analyzed whether these reads were enriched in specific regions, as indicated by read coverage along the chromosomes. With the 0.5 Gb dataset, the genome coverage with a read depth of at least 1 × was 16.6% ± 0.6% for AIO-seq, decreasing to 6.5% ± 0.2% for Panel A and 11.7% ± 0.2% for Panel B (Fig. 4I). In contrast, for regions covered with at least 3 × depth, the percentage for iRAD-seq was greater than that for AIO-seq. This trend was consistent across different data volumes from 0.5–2.0 Gb, with the differences between iRAD-seq and AIO-seq becoming more pronounced as the data volume increased (Fig. 4I-L). Between Panel_A and Panel_B, the efficiency of the reduced representation of Panel_A is greater than that of Panel_B (Fig. 4I-L). This result is consistent with the results of the digestion efficiency analysis (Fig. 4E-H). These results indicated that iRAD-seq exhibited a significant enrichment effect compared with whole genome resequencing, and that different RE panels can achieve a controllable and predictable level of genome representation.

In line with this, the number of SNPs increased with more sequencing data, and as the efficiency of reduced representation improved, the rate of increase decreased (Fig. 4M). This is because increasing amounts of data are more easily enriched in simplified genomic regions. Consequently, iRAD-seq can obtain more high-quality SNPs with a low data volume. Furthermore, analysis of the depth of the SNP loci revealed a similar distribution pattern to that of coverage (Fig. 4I-L, N and Additional file 3: Figs. S10A-S10C). With the 1.0 Gb dataset, iRAD-seq showing significantly greater number of SNPs after > 2 × and Panel_A significantly surpassed Panel_B after > 4 × (Fig. 4N). Both the 0.5 Gb、1.5 Gb and 2.0 Gb datasets showed similar patterns (Fig. 4N and Additional file 3: Figs. S9B and S9C). This result suggests that iRAD, relying on high-depth SNPs, can provide more accurate genetic analysis. In addition, with the 0.5 Gb dataset, the stability of the SNP quantity in iRAD is significantly better than that in AIO-seq. This result indicates that iRAD-seq exhibits better stability with a low dataset. The SNPs identified from the three methods were evenly distributed throughout the genome (Additional file 4: Fig. S11, Additional file 5: Fig. S12 and Additional file 6: Fig. S13). Collectively, these results suggest that iRAD-seq can effectively reduce genome representation, and that different REs can lead to varying degrees of reduction. The efficiency of reduced representation appears to correlate positively with the results of in silico digestion.

**Fig. 5:**
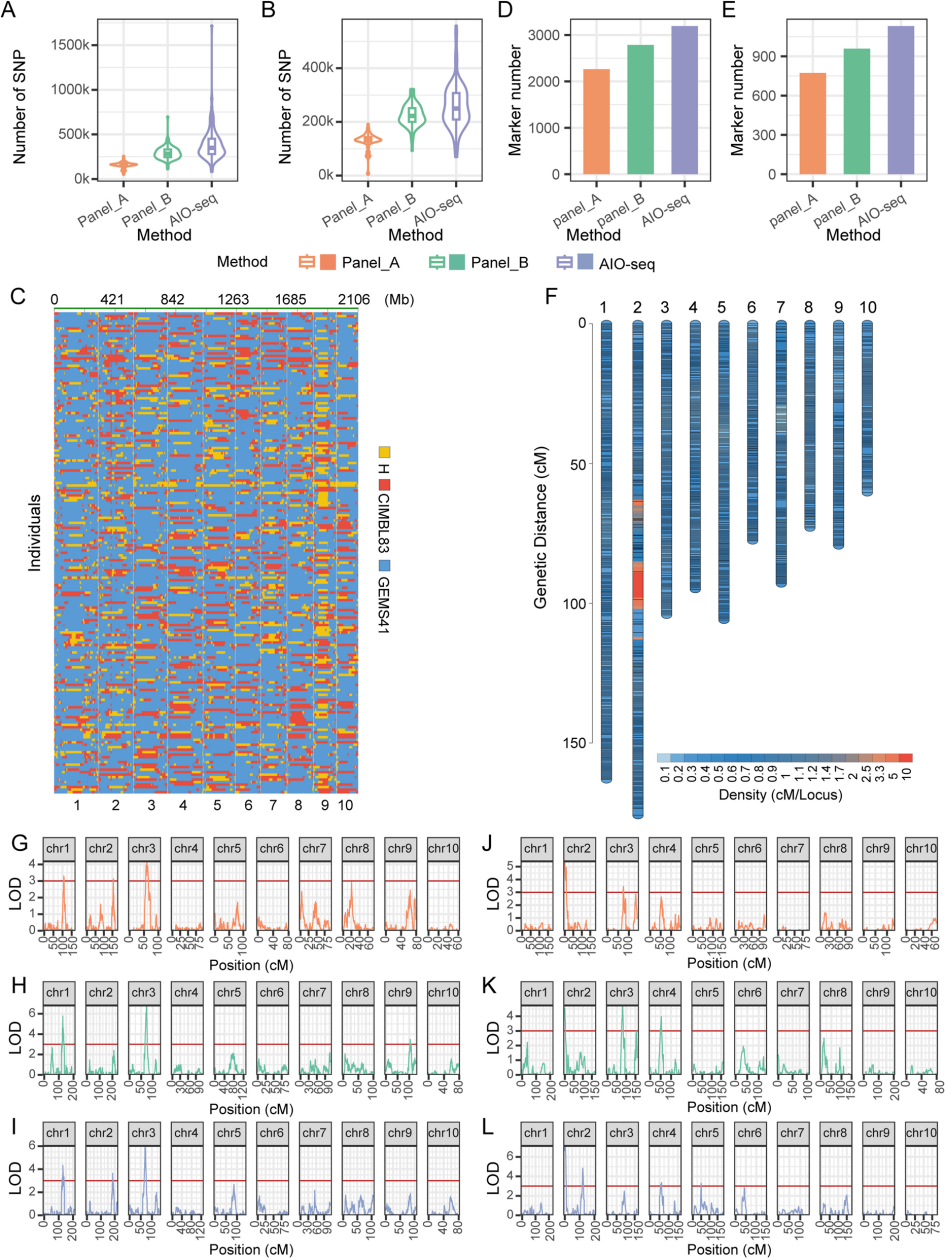
Application of iRAD-seq for maize genetics research. **A**-**B** Number of SNPs identified by different sequencing strategies in the CIMBL83/GEMS41 population (**A**) and CML496/GEMS41 population (**B**). For boxplots, the center line is the median; box limits represent upper and lower quartiles. **C** Construction of a recombination bin map for the CIMBL83/GEMS41 population with Panel_A. **D**-**E** Number of bin markers in CIMBL83/GEMS41 (**D**) and CML496/GEMS41 (**E**) by different sequencing strategies. **F** A high-density genetic map of CIMBL83/GEMS41 population with Panel_A. **G**-**L** LOD values of the quantitative trait locus (QTL) results for leaf angle (LOD value > 3) by different sequencing strategies in the CIMBL83/GEMS41 population (**G**-**I**) and CML496/GEMS41 population (**J**-**L**). Different methods use different REs (Panel_A: *Mse*I, *Msp*I and *Alu*I; Panel_B: *Mse*I, *Msp*I and *Hind*III; AIO-seq: none)

Across all lines of CIMBL83/GEMS41 and CML496/GEMS41, we identified 6,582,817 SNPs between the parents of CIMBL83/GEMS41 and 6,662,089 SNPs between the parents of CML496/GEMS41. On average, iRAD-seq with Panel_A, Panel_B, and AIO-seq yields 154,901 ± 32,878, 289,627 ± 67,365 and 386,207 ± 173,087 SNPs per line in CIMBL83/GEMS4 and 127,874 ± 32,039, 223,823 ± 41,585 and 260,258 ± 84,135 SNPs in CML496/GEMS41 (Fig. 5A, B and Additional file 8: Table S5). After genotyping, with these SNPs, we obtain 4465 (per lines 23), 4794 (25), and 4616 (24) breakpoints in CIMBL83/GEMS41 and 1137 (17), 1272 (19), and 1278 (19) in CML496/GEMS41 (Fig. 5C and Additional file 7: Figs. S14A-S14E). We detected 2261, 2785, and 3193 bin markers in CIMBL83/GEMS41 and 773, 958, and 1131 in CML496/GEMS41 (Fig. 5D, E and Additional file 8: Table S6). Using bin markers, we constructed linkage maps. We obtained 1027.5 cM, 1315.8 cM and 1314.8 cM genetic maps for CIMBL83/GEMS41 and 1299.1 cM, 1505.7 cM and 1682.1 cM genetic maps for CML496/GEMS41 (Fig. 5F, Additional file 7: Figs. S14F-S14J and Additional file 8: Table S6). Finally, we conducted quantitative trait locus (QTLs) mapping in combination with phenotypic (Additional file 7: Figs. S15A and S15B), and the results could be used to map the same main-effect QTLs (Fig. 5G-L and Additional file 8: Table S7). For example, in the CIMBL83/GEMS41 population, we successfully mapped a major QTL on chromosome 3, which included the previously identified *lg2* gene (Fig. 5G-I) [17]. In CML496/GEMS41, we mapped two major QTLs on chromosome 2, which included the previously identified *ibh1* and *lg1* genes (Fig. 5J-L) [18, 19]. Additionally, iRAD-seq identified a QTL that was not identified by AIO-seq, which included the previously identified *na1* gene in CML496/GEMS41 [20]. In general, the iRAD-seq method can perform accurate genotyping, high-density genetic mapping and major QTL mapping.

## Discussion

Genome-wide marker-assisted selection (MAS) breeding can significantly increase and accelerate the breeding process. Among various whole-genome genotyping methods, RAD-seq stands out as one of the most widely used techniques due to its versatility. It is applicable to a broad range of species, including non-model organisms, without the upfront investment required for probe/primer design and synthesis in multiple PCR and target enrichment methods [8, 21, 22]. RAD-seq, a form of RRS, uses REs to reduce the complexity of the genome, resulting in cost-effective sequencing while still providing high-quality data on genomic variation. However, for practical use in breeding programs, particularly in developing countries and for small- and medium-sized breeding companies, reducing per-sample costs remains a key objective for the development of WGG methods. RAD-seq and its derivatives employ various REs to digest the genome, followed by sequencing library preparation using adaptor ligation PCR to capture genomic sequences near RE sites [5, 11, 23].

The RAD-seq protocol fundamentally involves selecting a portion of the genome for library preparation. Typically, existing RAD-seq methods follow a two-step process: “select fragments first, then prepare library.” In these methods, the focus is on the positive selection of genomic fragments flanking the recognition sites of restriction enzymes for sequencing. In contrast, the iRAD-seq method we introduce here also selects a portion of the genome for library preparation, but differs in two important aspects. First, our approach involves “preparing library first, then selecting”. Second, instead of positive selection, we employ a negative selection strategy that targets fragments not flanking the recognition sites of the restriction enzymes.

Our innovative iRAD-seq method offers two key advantages over traditional approaches, distinguished by its simple protocol and high throughput for library preparation. First, iRAD-seq leverages the advantages of the Tn5 transposase for genome fragmentation and adaptor ligation, significantly reducing the process to just a 10-min reaction with a single enzyme. This contrasts with other RAD-seq methods that typically require restriction enzymes for genome fragmentation and T4 ligase for adaptor ligation, which increases both hands-on time and total experimental time. Second, following Tn5-based library construction, each sample is tagged with a unique barcode, allowing for the pooling of up to 96 libraries. These pooled libraries are then digested with restriction enzymes for the “fragment selection” step. In comparison, other RAD-seq methods can typically pool no more than 12 samples, or may not allow pooling at all. This gives iRAD-seq a much higher throughput than traditional RAD-seq methods. Third, although iRAD-seq is fundamentally a resequencing library, it selects only approximately 10% of the whole genome. Consequently, the bioinformatics analysis follows the same process as for whole-genome sequencing libraries, with the only difference being that the data volume is reduced to around 10%. This reduction simplifies the analysis process and results in significant savings in computational resources.

The improvements and innovations introduced by the iRAD-seq protocol have yielded significant benefits. Not only has the protocol reduced the consumption of reagents, but its streamlined workflow also makes it suitable for automation using liquid handling stations, thereby increasing throughput and reducing labor costs.

We conducted a rough comparison of the reagent costs for processing 96 samples using iRAD-seq versus traditional RAD-seq methods (Additional file 2: Table S8). For traditional RAD-seq, the primary costs are associated with restriction enzyme digestion and adaptor ligation. In contrast, the main cost for iRAD-seq comes from the Tn5 transposase-based library preparation. However, the pooling of samples prior to restriction enzyme digestion significantly reduces the need for restriction enzymes, DNA purification, and library quantification in the subsequent steps. Overall, while iRAD-seq relies on commercially available Tn5 transposase kits, which can be more expensive, the cost advantage over other RAD-seq methods may not be substantial for most labs. On the other hand, iRAD-seq uses only a single enzyme, Tn5 transposase, for both DNA fragmentation and adaptor ligation. Given the widespread use of Tn5 in the development of novel genomic research methods, several protocols for expressing homemade Tn5 transposase are already available [24–27]. Utilizing a self-made version of Tn5 can reduce the cost of library construction by over 80%, significantly enhancing the cost-effectiveness of iRAD-seq, especially for laboratories handling large sample volumes (Additional file 8: Table S8).

The simplified experimental workflow of iRAD-seq makes it highly suitable for automation using a liquid handling workstation. We have established the complete iRAD-seq protocol on a liquid handling system (Additional file 1: Fig. S16). With our setup, 960 samples can be processed in two days by one person, from the leaf sample to the sequence-ready iRAD-seq library. When preparing NGS libraries using transposases, the distribution of DNA fragment sizes is sensitive to the ratio of transposase to input DNA. To achieve optimal library fragmentation, several strategies have been proposed. The first and most straightforward strategy involves measuring the DNA concentration of the extracted samples and then adding varying volumes of water to normalize the concentration across all samples. This process can be cumbersome when handling large numbers of samples manually. A liquid handler with either individual channels or an independent 8-channel system can automate this step, but throughput remains limited, and some manual intervention is still required. During our iRAD-seq experiments, we employed an automated nucleic acid concentration analysis and normalization workstation (Automatic Nucleic Acid Concentration Analysis and Normalization Workstation, HC Scientific, Chengdu, China) for DNA concentration normalization. This instrument is specifically designed for DNA normalization during NGS library construction, using a stacker system that can complete concentration measurements and normalization for 10 96-well plates in 260 min, with no manual intervention required. This significantly improved the efficiency of iRAD-seq library construction. The second strategy involves engineering the Tn5 transposase to enhance its tolerance to varying ratios of input DNA and enzymes during tagmentation, thereby reducing the stringent need for DNA concentration normalization [26, 27]. The third strategy involves conjugating Tn5 transposase directly to magnetic beads, enabling it to bind a fixed amount of DNA and produce a normalized, sequencing-ready library [28]. This approach is suitable for a wide range of DNA input quantities and largely eliminates the need for the most cumbersome step of DNA quantification and normalization. We believe this approach is the most effective way to minimize the impact of the Tn5 to input DNA ratio on the final DNA fragment distribution. If the iRAD-seq method we developed can eventually incorporate bead-linked transposomes, it will further decrease the dependence on costly liquid handling workstations and streamline the experimental workflow.

## Conclusions

The development of iRAD-seq represents a significant advancement in RRS methods for whole-genome genotyping. Leveraging Tn5 transposase for simultaneous DNA fragmentation and adapter ligation, this method drastically simplifies library construction, reduces hands-on time, and enhances automation compatibility. Validated in maize and rice, iRAD-seq achieves consistent genome-wide SNP distributions, with in silico predictions aligning closely with empirical data, confirming its precision in excluding restriction-associated fragments. Customizable enzyme panels enable flexible genome representation, balancing marker density for diverse applications.

iRAD-seq demonstrated practical utility in resolving maize germplasm population structure and mapping agronomically relevant QTLs, underscoring its reliability through high technical reproducibility. By integrating cost-effective Tn5 protocols and scalable automation, it addresses bottlenecks in large-scale genotyping, particularly for resource-limited breeding initiatives. Collectively, iRAD-seq bridges high-throughput sequencing demands and agricultural genomics, offering a streamlined, efficient platform for molecular breeding and functional genomics.

## Methods

### Plant materials and DNA extraction

The samples of maize (*Zea mays* L. *cv*. B73) and rice (*Oryza sativa* ssp*. japonica cv.* Nipponbare) were obtained from the Agricultural Genomic Institute at ShenZhen, Chinese Academy of Agricultural Sciences (AGIS, CAAS). A panel consisting 139 natural maize lines, including sweet corn (55) and field corn from China and USA (84). These inbred lines were received as a gift from Dr. Lu Hong (AGIS, CAAS). Two maize (*Zea mays*) populations were obtained from a previous study. The first population, BC_1_F_4_ (193 lines), was derived from a cross between GEMS41 and CIMBL83, followed by backcrossing to GEMS41 and selfing for four generations. The second population, BC_2_F_4_ (68 lines), was derived from a cross between GEMS41 and CML496, followed by backcrossed to GEMS41, and selfed for four generations [29].

Genomic DNA was extracted from the leaves of each accession using the modified CTAB method [30]. NanoDrop2000 (Thermo Fisher Scientific) and Qubit™ 4.0 Fluorometer (Invitrogen, NY, USA) were used to quantify the DNA. DNA samples were inspected on 1% agarose gels for the presence of lower molecular weight DNA. DNA normalization utilizing MetricAnalyzer 3430S (Automatic Nucleic Acid Concentration Analysis and Normalization Workstation, HC Scientific, Chengdu, China).

### In silico digestions of Genome

The reference genomes of maize (B73_v4) [31], rice (ZhenShan97) [32], soybean (Glycine_v2.0) [33], wheat (CS_v1.0) [34], cow (bosTau_v9) [35] and pig (susScr_v11.1) [36] were downloaded from the NCBI database (Additional file 2: Table S1). The Perl script fa2cmap_multi_color.pl from Solve-V3.5 [37] was used to find the recognition site of REs in the genomes. A self-written Perl script find_fragments_length.pl was used to calculate the positions and lengths of fragments (a sequence between two recognition sites of REs) in the genome that exceed a threshold length.

### Library preparation of AIO-seq and sequencing

50 ng gDNA was digested with 2.5 μL Tn5 transposase from the TruePrep® DNA Library Prep Kit V2 for Illumina® (Vazyme Biotech, Nanjing, China, Cat. TD501-02) for 10 min at 55 ℃ in an 8 μL reaction volume. These fragment products were subjected to PCR amplification with 5 μL 10 × TAB buffer, 1 μL TAE, 3 μL PPM, 0.5 mM P5 and P7 primers in the same tube, TAB buffer and TAE from the TruePrep Amplify Enzyme Kit (Vazyme Biotech, Nanjing, China, Cat. TD601-01). The thermocycling program was as follows: chain displacement at 72 °C; pre-denaturation at 98 °C for 2 min; five cycles of 98 °C for 30 s, 60 °C for 30 s, and 72 °C for 3 min; and a final extension at 72 °C for 5 min. The amplification products were purified with 1.2 × beads (Vazyme Biotech, Nanjing, China, Cat. N411-02). All libraries were pooled according to equal amounts of products in one tube using the AIO-seq method [16]. The quality inspection of the library was performed on BiOptic Qsep100 Bio-Fragment Analyzer (Bioptic, C100100). Fragments from 430 to 780 bp was obtained using PippinHT (Sage) and sequenced on NovaSeq 6000 platform (Illumina, San Diego, CA, USA) by the Berry Genomics Company (Beijing, China).

### Library preparation of iRAD-seq and sequencing

The library preparation is identical to AIO-seq. However, to delete the sequence containing the RE recognition site, the pooled library was digested by REs before sorting. The reaction was kept at 37℃ with 1 μg DNA from the pooled library, 5 μL 10 × Cutsmart buffer and 10 U of each RE in a 50 μL reaction system. The digested products were subsequently purified using Cycle-Pure kit (OMEGA). After quality inspection and sorting, the library was sequenced on the sequencer.

### Sequence quality checking and SNPs calling

The quality of the data was initially assessed using FastQC (V0.11.8) [38]. The data were trimmed using Trimmomatic (V0.39) [39] to remove adapter sequences and low-quality terminals. Duplicated data were then identified and removed using FastUniq (V1.1) [40]. The high-quality data were mapped to the reference genome (B73_V4 [31] or ZhenShan97 [32]) using BWA-MEM [41]. We used Samtools [42] and GATK (V4.1.8.1) [43] to sort BAM files and mark duplicated reads. We performed variant calling using the GATK with the HaplotypeCaller command and default settings. We filtered the SNP information based on the modified parameters (QD < 2.0, QUAL < 30.0, SOR > 3.0, FS > 60.0, MQ < 40.0, MQRankSum < −12.5, ReadPosRankSum < −8.0) by GATK.

### Reproducibility and reliability evaluation

To ensure the reproducibility and reliability of iRAD-seq method, we performed iRAD-seq on maize (B73) and rice (Nip) using Panel_A (*Alu*I, *Mse*I, *Msp*I) and Panel_B (*Hind*III, *Mse*I, *Msp*I), with four biological replicates. Using the sample module in seqkit [44], we randomly subsampled sequencing data at different scales (maize: 0.5 Gb, 1.0 Gb, 2.0 Gb, and 4.0 Gb; rice: 0.25 Gb, 0.5 Gb, 1.0 Gb, and 2.0 Gb) for downstream analysis. We used the bamqc tool in QualiMap [45] to analyze the distribution of genome coverage. We performed sliding window statistics with a window size of 100 kb to calculate the average depth across each window using bedtools [46]. Then, we calculated Spearman's rank correlation coefficient (SRCC) between all pairs of duplicates and plotted the correlation coefficient graph using R package corrplot [47]. Besides, we obtain high-quality SNP information through the GATK pipeline. The R package VennDiagram [48] was used to draw Venn diagrams among repeats.

### Phylogenetic relationship and population structure analysis

VCFtools (V0.1.13) [49] was used to select a subset of high-quality SNPs for subsequent analyses for maize natural lines using the following options: ‘–max-missing 0.8 –maf 0.05’. The high-quality SNP files for population structure analysis were transformed into plink files using VCFtools (V0.1.13) [49]. Then, we perform principal component analysis (PCA) using Plink (V1.9) [50], and PCA diagram was drawn using the R package ggplot2 [51]. We used VCF2Dis (https://github.com/BGI-shenzhen/VCF2Dis) to calculate a P distance matrix based on all the SNPs. The distance matrix was further calculated by FastME (V2.0) [52] to generate an unroot neighbor-joining (NJ) tree file with BIONJ method [53], and unroot tree diagram was drawn using R package ggtree [54].

### Population genetic analysis with BCF lines

A sliding window approach was used to find recombination breakpoints of the BC_1_F_4_ and BC_2_F_4_ lines. We used the Python 1 script to select homozygous SNP sites with polymorphism between parents. The Python 2 script can select SNP sites in progeny that are consistent with the SNP sites from the results of Python 1, and can also genotype SNPs. The R language and the python 3 and the python 4 scripts were used to find the recombination breakpoints, after which bin markers were obtained. Bin map plots were drawn using RectChr [55]. The calculation of genetic distance was performed using QTL IciMapping [56], and the QTL were statistic with WinQTLCart (v2.5) [57]. Genetic map and LOD diagrams were drawn by the R package LinkageMapView and ggplot2, respectively. Python and R scripts are available at Zenodo [58].

## Supplementary Information

Additional file 1: Figure S1. The percentage of the genome covered by fragments larger than 300 bp after in silico digestion. Figure S2. Distribution of fragments after enzymatic digestion.Genome coverage analysis of fragments in soybean and wheat.The number of fragments in soybean and wheat. Five different panel of REs were selected for in silico digestion of the genome. Figure S3. Chromosome distribution of fragments after enzymatic digestion. Different genomes were digested by five different panel of REs. The size of sliding window is 10 kb. Red, high density; green, low density.

Additional file 2: Figure S4. Evaluation of RE efficiency.Proportion of reads without RE recognition sites with Panel_A and Panel_B in maize and rice.Proportion of reads without each RE recognition sites with Panel_A and Panel_B in maize and rice. Panel_A: MseI, MspI and AluI; Panel_B: MseI, MspI and HindIII. Figure S5. Consistency of coverage among 4 repeats when using varying sequencing data sizes. Pairwise Spearman correlation of the mean coverage between replicates with 100 kb windows. Panel_A: MseI, MspI and AluI; Panel_B: MseI, MspI and HindIII. Figure S6. The overlap of SNPs among 4 repeats when using varying sequencing data sizes. Panel_A: MseI, MspI and AluI; Panel_B: MseI, MspI and HindIII. Figure S7. Application of iRAD-seq in maize germplasm.Sequencing yields of 132 maize germplasms.1812 high-quality SNPs of 139 maize germplasms widespread in the genome. Panel_A: MseI, MspI and AluI.

Additional file 3: Figure S8. Sequencing yields of the maize genetic population.Sequencing yields of different sequencing strategies in the CIMBL83/GEMS41 population.Sequencing yields of different sequencing strategies in the CML496/GEMS41 population. For boxplots, center line is the median; box limits represent upper and lower quartiles. Different methods use different REs. Figure S9. iRAD significantly reduces the number of reads containing restriction enzyme recognition sites.Proportion of reads without AluI recognition sites in the CIMBL83/GEMS41 populationand CML496/GEMS41 populationwith Panel_A, Panel_B and AIO-seq.Proportion of reads without HindIII recognition sites in the CIMBL83/GEMS41 populationand CML496/GEMS41 populationwith Panel_A, Panel_B and AIO-seq. Different methods use different REs. Figure S10. iRAD-seq can enhance the depth of the obtained SNPs.The number of SNPs at different depths. The data used in the experiment was 0.5 Gb, 1.5 Gb, and 2.0 Gb. SNP number at different depths. Log10: n is the number of SNPs. For boxplots, center line is the median; box limits represent upper and lower quartiles. Employ the t-test to assess differences in different method, p < 0.0001. Different methods use different REs.

Additional file 4: Figure S11. Chromosome distribution of SNPs when using varying sequencing data sizes with Panel_A. The size of the sliding window is 1 Mb. Red, high density; green, low density.

Additional file 5: Figure S12. Chromosome distribution of SNPs when using varying sequencing data sizes with panel_B. The size of the sliding window is 1 Mb. Red, high density; green, low density.

Additional file 6: Figure S13. Chromosome distribution of SNPs when using varying sequencing data sizes with AIO-seq. The size of the sliding window is 1 Mb. Red, high density; green, low density.

Additional file 7: Figure S14. Bin maps and genetic maps of iRAD-seq.Construction of recombination bin maps illustrating recombination events in the CIMBL83/GEMS41and CML496/GEMS41 populationswith different sequencing strategies.High-density genetic maps of the CIMBL83/GEMS41and CML496/GEMS41 populationwith different sequencing strategies. Different methods use different REs. Figure S15. Phenotypic distribution of the leaf angle.Distribution of 193 maize lines from CIMBL83/GEMS41.The distribution of 68 maize lines from CML496/GEMS41. Figure S16. iRAD-seq workflow integrated with a liquid handling automation system. The estimated time required is based on the processing of 960 samples.

Additional file 8: Table S1. Genome information of different species for in silico digestion. Table S2. Sequencing information and statistics for maizeand rice. Table S3. Sequencing information and statistics for a germplasm population of 132 maize lines. Table S4. Information of genotype from maize germplasm for phylogenetic analysis. Table S5. Sequencing information and statistics for two BCF4 populations of maize. Table S6. Summary of the genetic map for the maize BCF4 population. Table S7. Summary of all quantitative trait lociidentified. Table S8. Comparison of performance between iRAD-seq and other RAD methods.

## Data Availability

The raw sequence data reported in this paper have been deposited in the Genome Sequence Archive [59] in National Genomics Data Center [60], China National Center for Bioinformation/Beijing Institute of Genomics, Chinese Academy of Sciences (GSA: CRA024789) that are publicly accessible at GSA [61], and are also available in the NCBI Sequence Read Archive (SRA) under accession number PRJNA1216733 [62]. The custom scripts and codes used in this study are available at Zenodo [58].

## Abbreviations

RRS: Reduced representation sequencing
RAD-seq: Restriction site-associated DNA sequencing
iRAD-seq: Inverse Restriction site-associated DNA sequencing
SNP: Single-Nucleotide Polymorphism
QTLs: Quantitative trait loci
dd-RAD: Double digest-RAD
RE: Restriction enzyme
AIO-seq: All-in-one sequencing
MAS: Marker-assisted selection
SRCC: Spearman's rank correlation coefficient
PCA: Principal component analysis
NJ: Neighbor-joining
SRA: Sequence Read Archive
