# Supplementary figures and images for: A novel method for effectively selecting fragments not associated with restriction sites for whole-genome genotyping
Source: BMC Biol. 2025 Oct 30;23:327. doi: 10.1186/s12915-025-02330-8 (PMC12573827; doi:10.1186/s12915-025-02330-8)

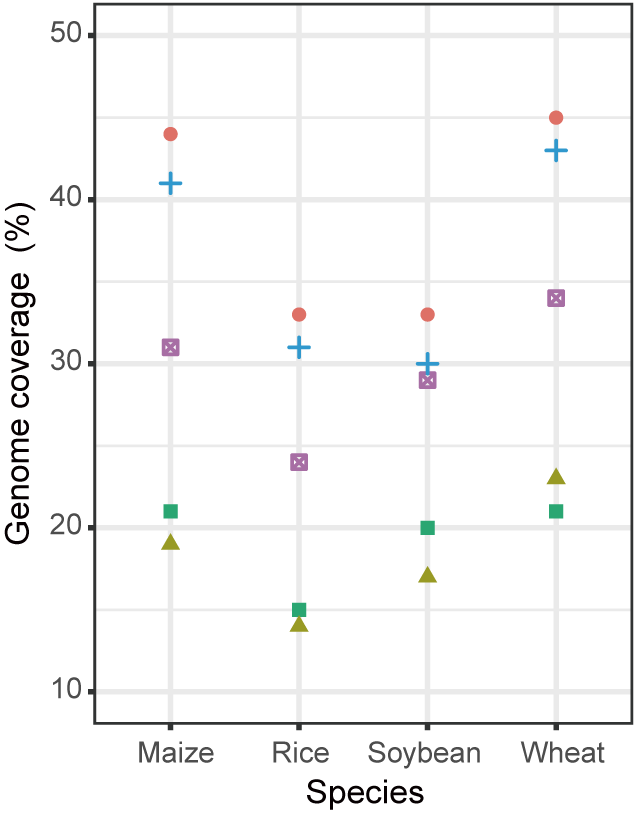

Supplement: Supplementary file 1 — Additional file 1: Figure S1. The percentage of the genome covered by fragments larger than 300 bp after in silico digestion. Figure S2. Distribution of fragments after enzymatic digestion.Genome coverage analysis of fragments in soybean and wheat.The number of fragments in soybean and wheat. Five different panel of REs were selected for in silico digestion of the genome. Figure S3. Chromosome distribution of fragments after enzymatic digestion. Different genomes were digested by five different panel of REs. The size of sliding window is 10 kb. Red, high density; green, low density. [file 12915_2025_2330_MOESM1_ESM.zip › Fig S1.tif]

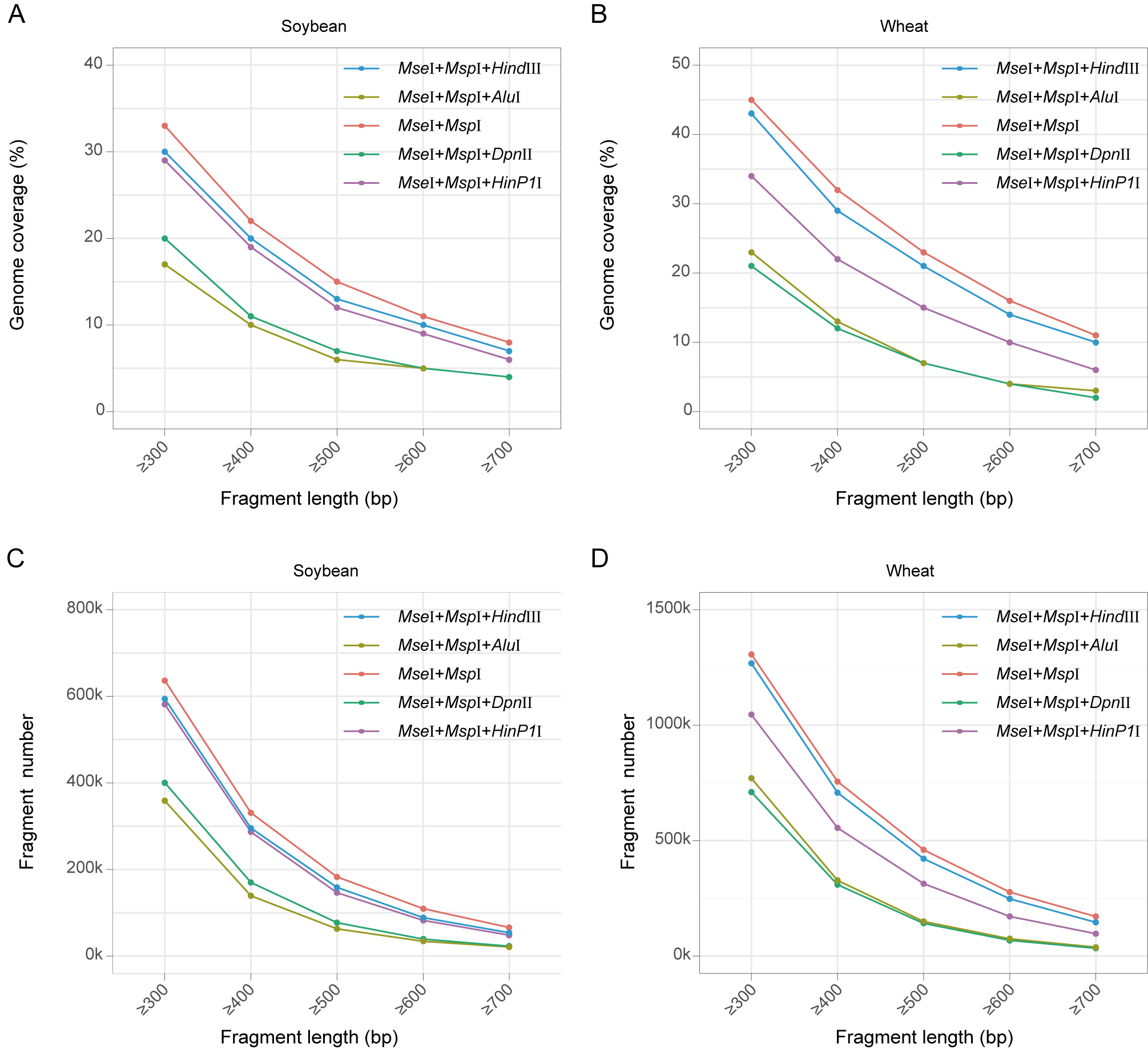

Supplement: Supplementary file 1 — Additional file 1: Figure S1. The percentage of the genome covered by fragments larger than 300 bp after in silico digestion. Figure S2. Distribution of fragments after enzymatic digestion.Genome coverage analysis of fragments in soybean and wheat.The number of fragments in soybean and wheat. Five different panel of REs were selected for in silico digestion of the genome. Figure S3. Chromosome distribution of fragments after enzymatic digestion. Different genomes were digested by five different panel of REs. The size of sliding window is 10 kb. Red, high density; green, low density. [file 12915_2025_2330_MOESM1_ESM.zip › Fig S2.tif]

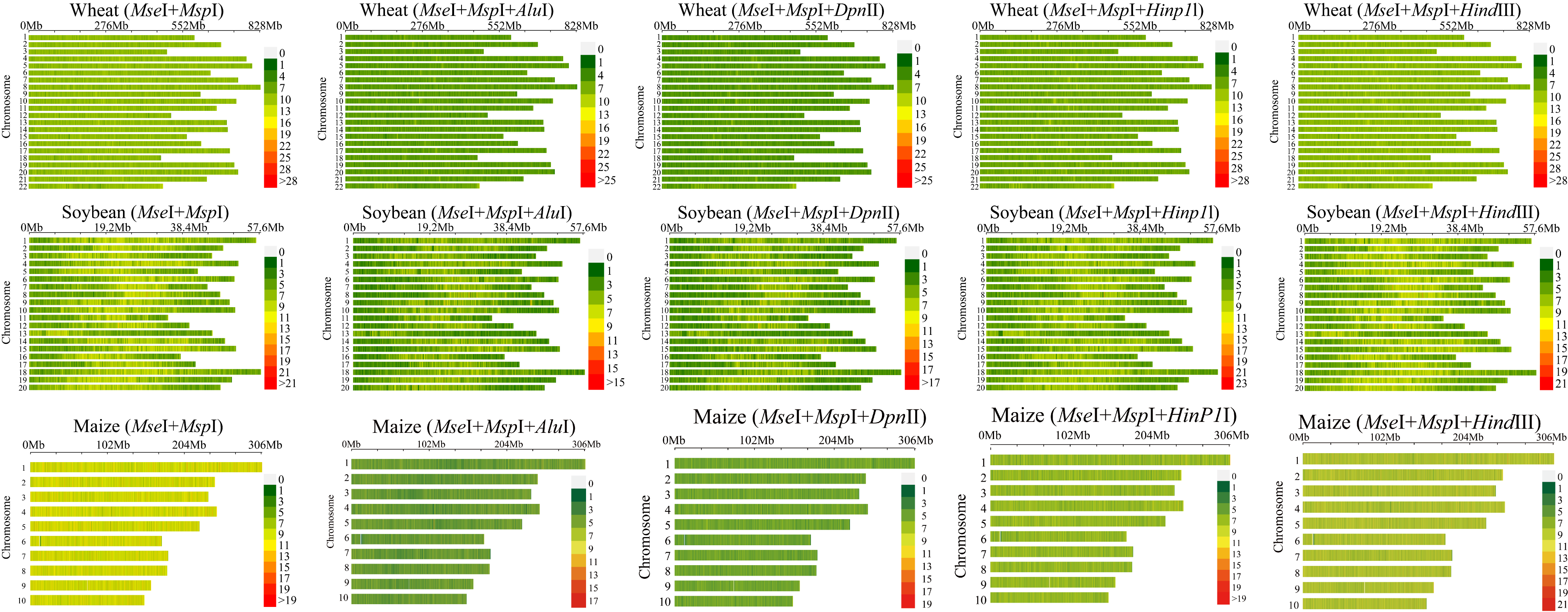

Supplement: Supplementary file 1 — Additional file 1: Figure S1. The percentage of the genome covered by fragments larger than 300 bp after in silico digestion. Figure S2. Distribution of fragments after enzymatic digestion.Genome coverage analysis of fragments in soybean and wheat.The number of fragments in soybean and wheat. Five different panel of REs were selected for in silico digestion of the genome. Figure S3. Chromosome distribution of fragments after enzymatic digestion. Different genomes were digested by five different panel of REs. The size of sliding window is 10 kb. Red, high density; green, low density. [file 12915_2025_2330_MOESM1_ESM.zip › Fig S3.tif]

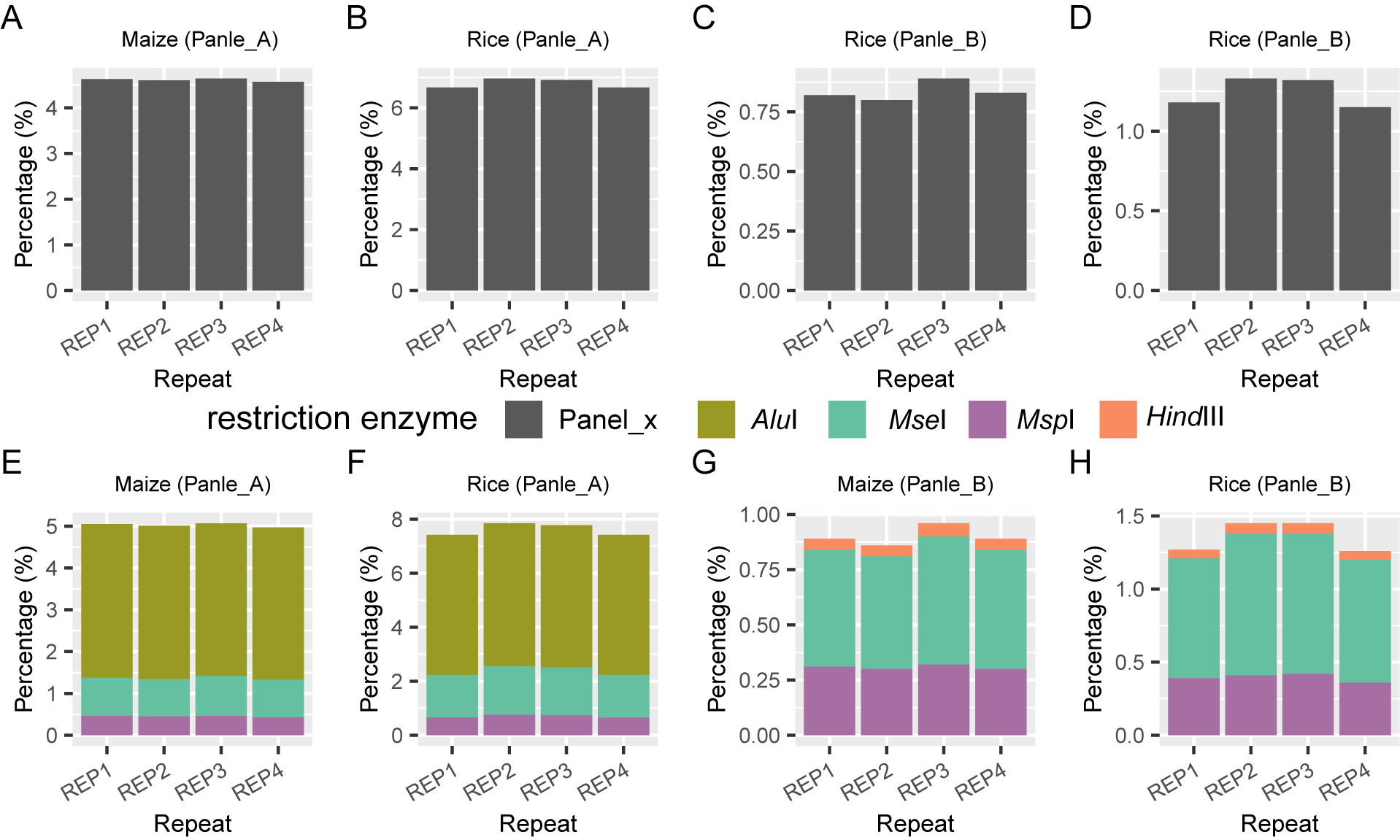

Supplement: Supplementary file 2 — Additional file 2: Figure S4. Evaluation of RE efficiency.Proportion of reads without RE recognition sites with Panel_A and Panel_B in maize and rice.Proportion of reads without each RE recognition sites with Panel_A and Panel_B in maize and rice. Panel_A: MseI, MspI and AluI; Panel_B: MseI, MspI and HindIII. Figure S5. Consistency of coverage among 4 repeats when using varying sequencing data sizes. Pairwise Spearman correlation of the mean coverage between replicates with 100 kb windows. Panel_A: MseI, MspI and AluI; Panel_B: MseI, MspI and HindIII. Figure S6. The overlap of SNPs among 4 repeats when using varying sequencing data sizes. Panel_A: MseI, MspI and AluI; Panel_B: MseI, MspI and HindIII. Figure S7. Application of iRAD-seq in maize germplasm.Sequencing yields of 132 maize germplasms.1812 high-quality SNPs of 139 maize germplasms widespread in the genome. Panel_A: MseI, MspI and AluI. [file 12915_2025_2330_MOESM2_ESM.zip › Fig S4.tif]

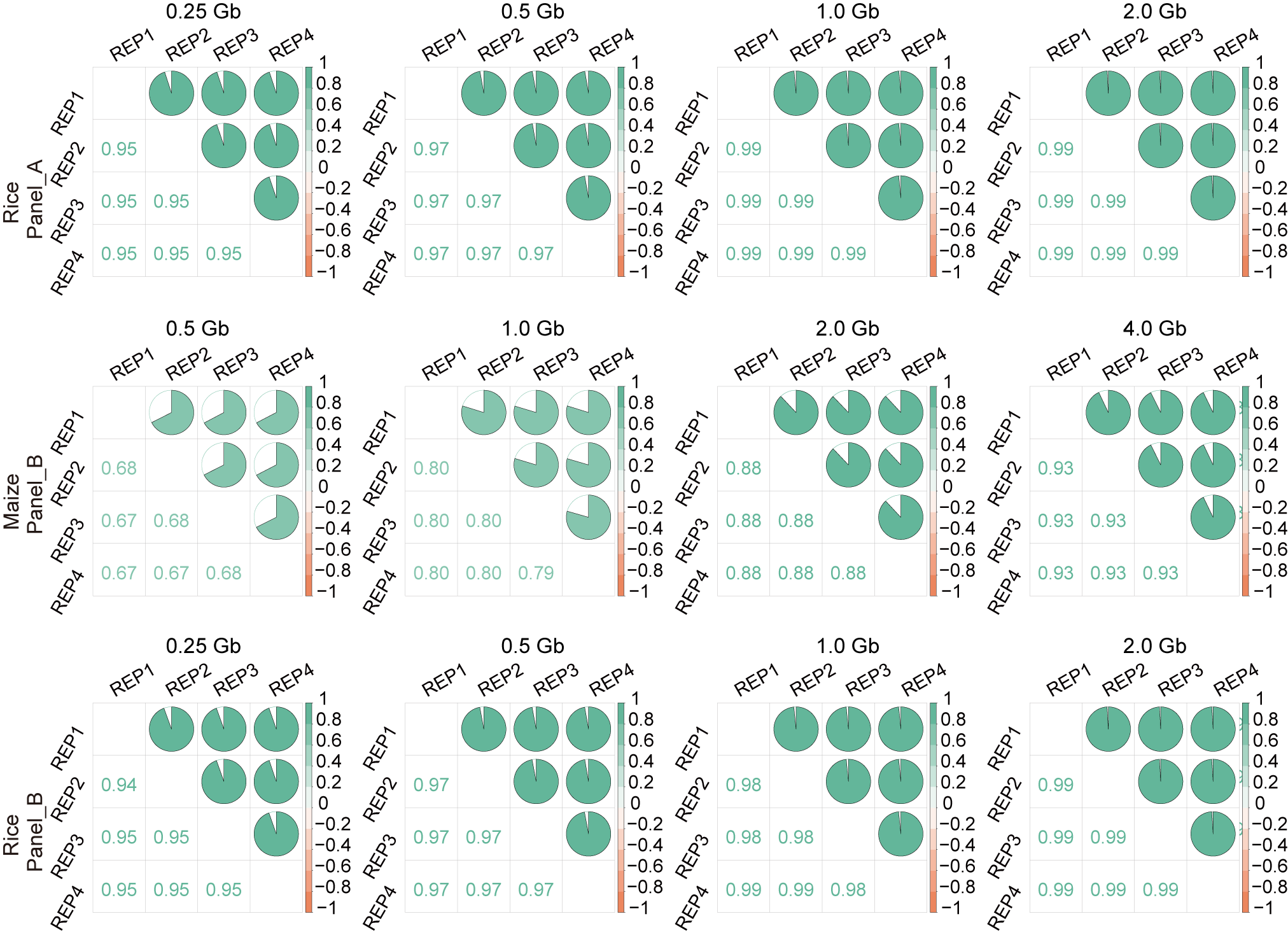

Supplement: Supplementary file 2 — Additional file 2: Figure S4. Evaluation of RE efficiency.Proportion of reads without RE recognition sites with Panel_A and Panel_B in maize and rice.Proportion of reads without each RE recognition sites with Panel_A and Panel_B in maize and rice. Panel_A: MseI, MspI and AluI; Panel_B: MseI, MspI and HindIII. Figure S5. Consistency of coverage among 4 repeats when using varying sequencing data sizes. Pairwise Spearman correlation of the mean coverage between replicates with 100 kb windows. Panel_A: MseI, MspI and AluI; Panel_B: MseI, MspI and HindIII. Figure S6. The overlap of SNPs among 4 repeats when using varying sequencing data sizes. Panel_A: MseI, MspI and AluI; Panel_B: MseI, MspI and HindIII. Figure S7. Application of iRAD-seq in maize germplasm.Sequencing yields of 132 maize germplasms.1812 high-quality SNPs of 139 maize germplasms widespread in the genome. Panel_A: MseI, MspI and AluI. [file 12915_2025_2330_MOESM2_ESM.zip › Fig S5.tif]

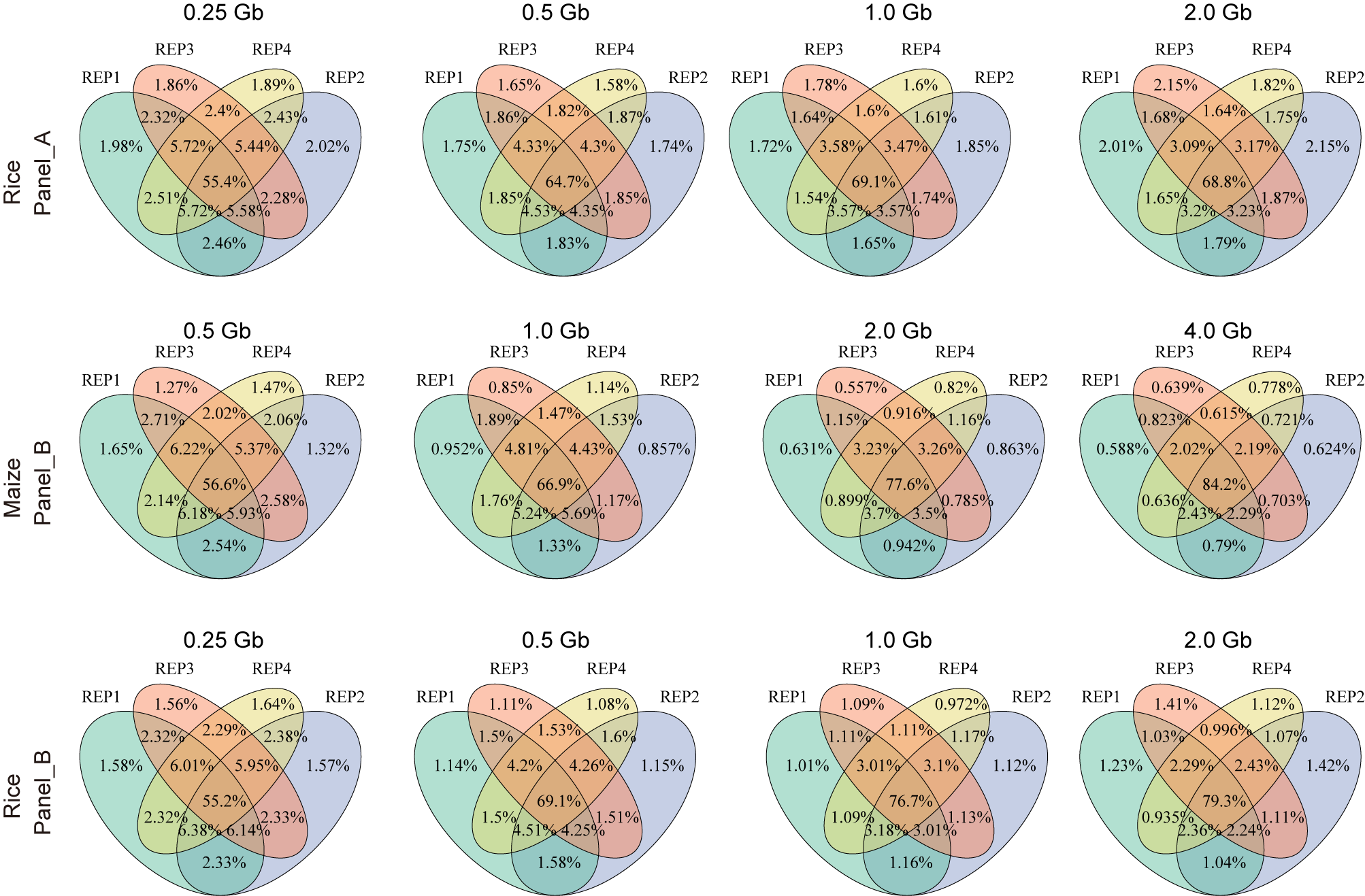

Supplement: Supplementary file 2 — Additional file 2: Figure S4. Evaluation of RE efficiency.Proportion of reads without RE recognition sites with Panel_A and Panel_B in maize and rice.Proportion of reads without each RE recognition sites with Panel_A and Panel_B in maize and rice. Panel_A: MseI, MspI and AluI; Panel_B: MseI, MspI and HindIII. Figure S5. Consistency of coverage among 4 repeats when using varying sequencing data sizes. Pairwise Spearman correlation of the mean coverage between replicates with 100 kb windows. Panel_A: MseI, MspI and AluI; Panel_B: MseI, MspI and HindIII. Figure S6. The overlap of SNPs among 4 repeats when using varying sequencing data sizes. Panel_A: MseI, MspI and AluI; Panel_B: MseI, MspI and HindIII. Figure S7. Application of iRAD-seq in maize germplasm.Sequencing yields of 132 maize germplasms.1812 high-quality SNPs of 139 maize germplasms widespread in the genome. Panel_A: MseI, MspI and AluI. [file 12915_2025_2330_MOESM2_ESM.zip › Fig S6.tif]

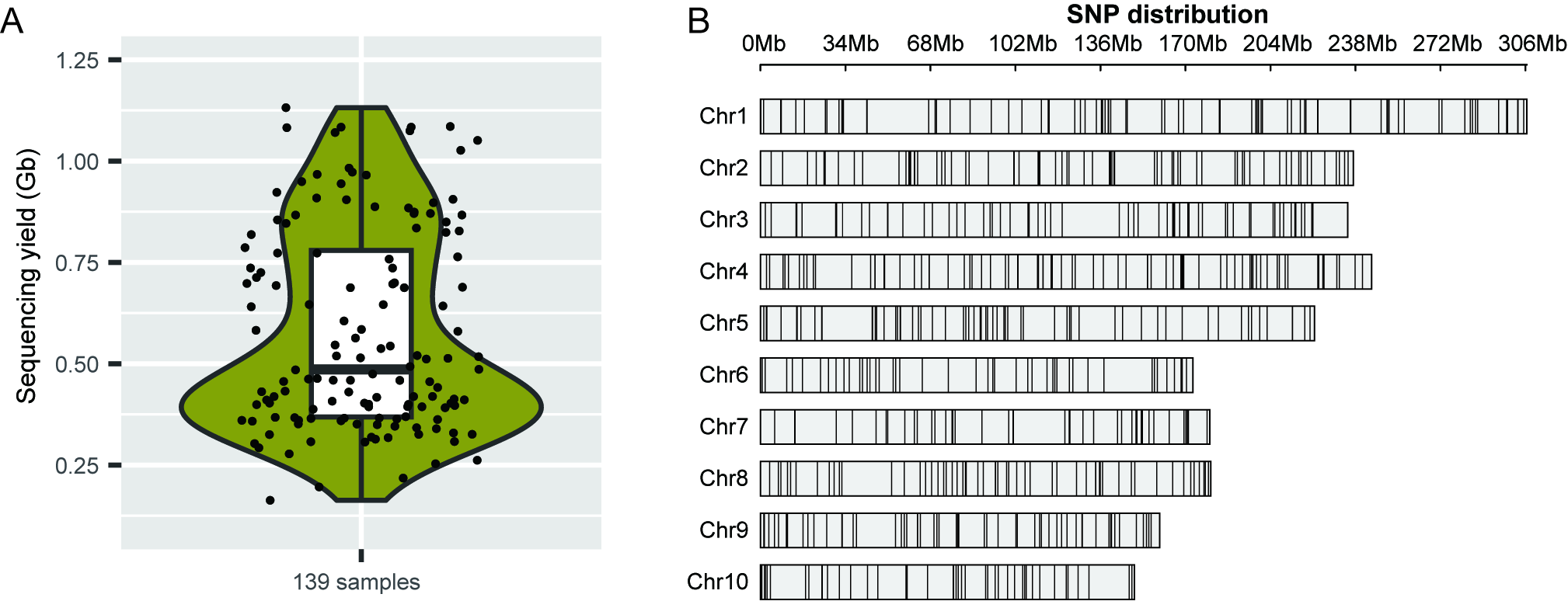

Supplement: Supplementary file 2 — Additional file 2: Figure S4. Evaluation of RE efficiency.Proportion of reads without RE recognition sites with Panel_A and Panel_B in maize and rice.Proportion of reads without each RE recognition sites with Panel_A and Panel_B in maize and rice. Panel_A: MseI, MspI and AluI; Panel_B: MseI, MspI and HindIII. Figure S5. Consistency of coverage among 4 repeats when using varying sequencing data sizes. Pairwise Spearman correlation of the mean coverage between replicates with 100 kb windows. Panel_A: MseI, MspI and AluI; Panel_B: MseI, MspI and HindIII. Figure S6. The overlap of SNPs among 4 repeats when using varying sequencing data sizes. Panel_A: MseI, MspI and AluI; Panel_B: MseI, MspI and HindIII. Figure S7. Application of iRAD-seq in maize germplasm.Sequencing yields of 132 maize germplasms.1812 high-quality SNPs of 139 maize germplasms widespread in the genome. Panel_A: MseI, MspI and AluI. [file 12915_2025_2330_MOESM2_ESM.zip › Fig S7.tif]

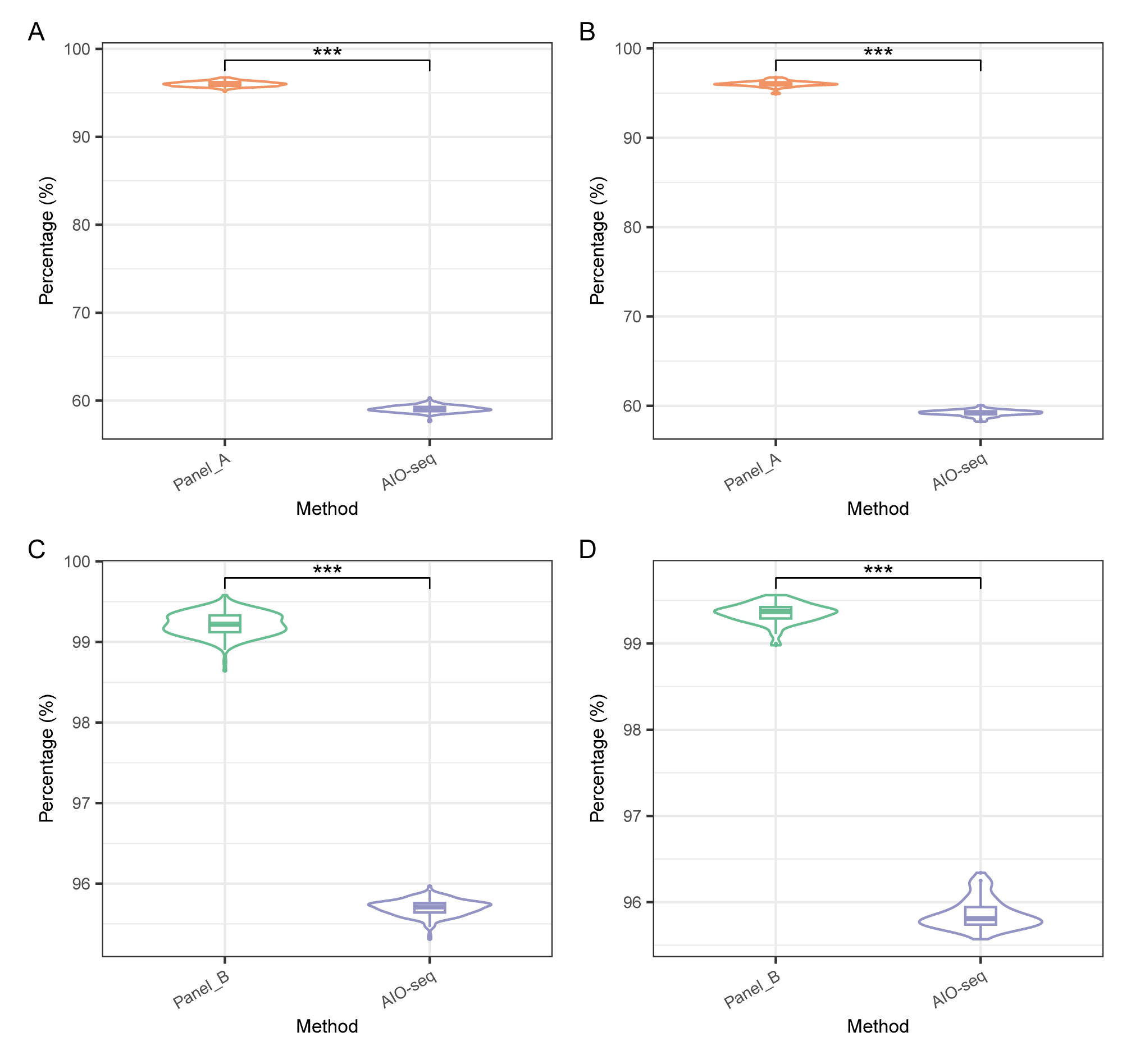

Supplement: Supplementary file 3 — Additional file 3: Figure S8. Sequencing yields of the maize genetic population.Sequencing yields of different sequencing strategies in the CIMBL83/GEMS41 population.Sequencing yields of different sequencing strategies in the CML496/GEMS41 population. For boxplots, center line is the median; box limits represent upper and lower quartiles. Different methods use different REs. Figure S9. iRAD significantly reduces the number of reads containing restriction enzyme recognition sites.Proportion of reads without AluI recognition sites in the CIMBL83/GEMS41 populationand CML496/GEMS41 populationwith Panel_A, Panel_B and AIO-seq.Proportion of reads without HindIII recognition sites in the CIMBL83/GEMS41 populationand CML496/GEMS41 populationwith Panel_A, Panel_B and AIO-seq. Different methods use different REs. Figure S10. iRAD-seq can enhance the depth of the obtained SNPs.The number of SNPs at different depths. The data used in the experiment was 0.5 Gb, 1.5 Gb, and 2.0 Gb. SNP number at different depths. Log10: n is the number of SNPs. For boxplots, center line is the median; box limits represent upper and lower quartiles. Employ the t-test to assess differences in different method, p < 0.0001. Different methods use different REs. [file 12915_2025_2330_MOESM3_ESM.zip › Fig S9.tif]

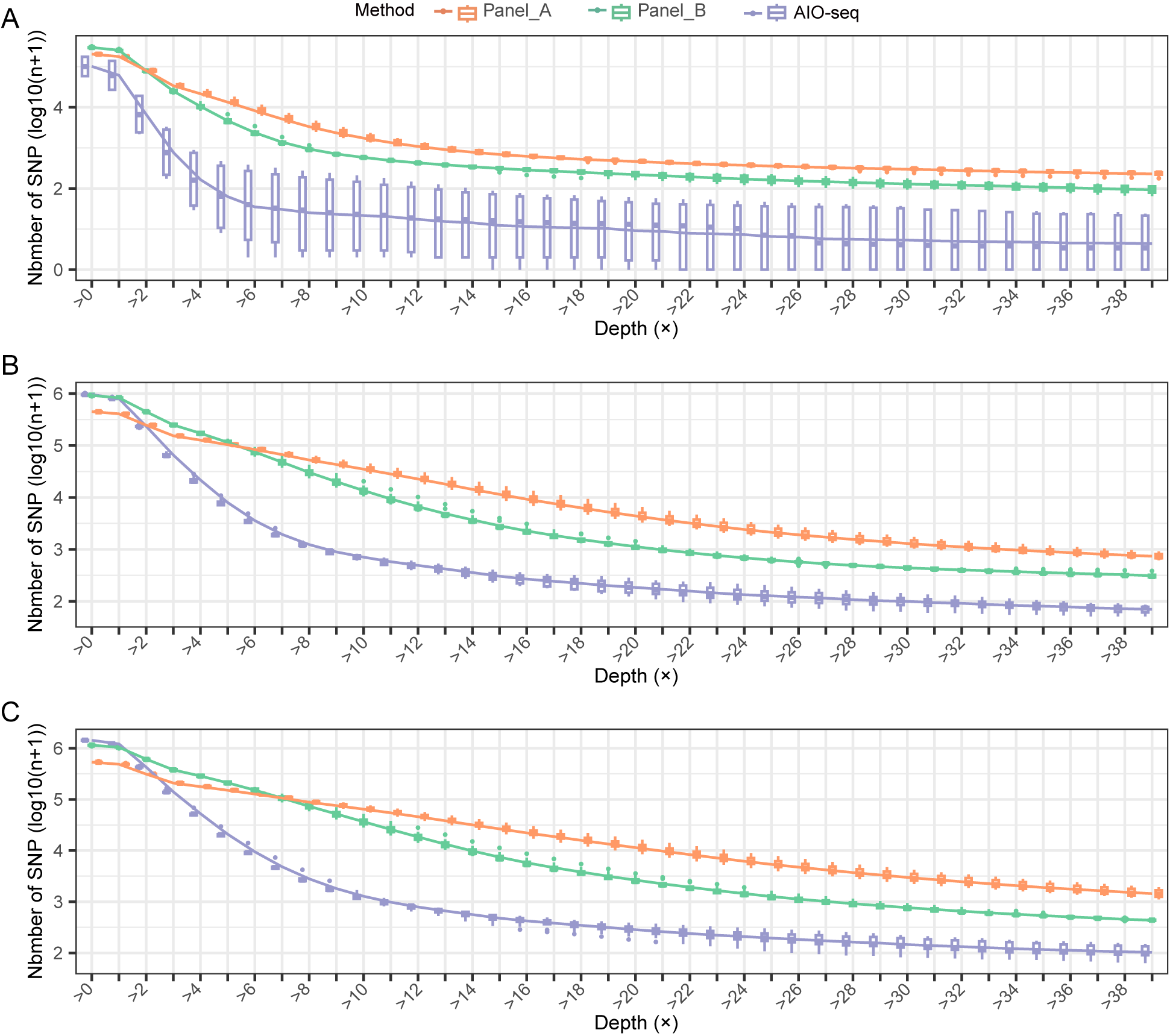

Supplement: Supplementary file 3 — Additional file 3: Figure S8. Sequencing yields of the maize genetic population.Sequencing yields of different sequencing strategies in the CIMBL83/GEMS41 population.Sequencing yields of different sequencing strategies in the CML496/GEMS41 population. For boxplots, center line is the median; box limits represent upper and lower quartiles. Different methods use different REs. Figure S9. iRAD significantly reduces the number of reads containing restriction enzyme recognition sites.Proportion of reads without AluI recognition sites in the CIMBL83/GEMS41 populationand CML496/GEMS41 populationwith Panel_A, Panel_B and AIO-seq.Proportion of reads without HindIII recognition sites in the CIMBL83/GEMS41 populationand CML496/GEMS41 populationwith Panel_A, Panel_B and AIO-seq. Different methods use different REs. Figure S10. iRAD-seq can enhance the depth of the obtained SNPs.The number of SNPs at different depths. The data used in the experiment was 0.5 Gb, 1.5 Gb, and 2.0 Gb. SNP number at different depths. Log10: n is the number of SNPs. For boxplots, center line is the median; box limits represent upper and lower quartiles. Employ the t-test to assess differences in different method, p < 0.0001. Different methods use different REs. [file 12915_2025_2330_MOESM3_ESM.zip › Fig S10.tif]

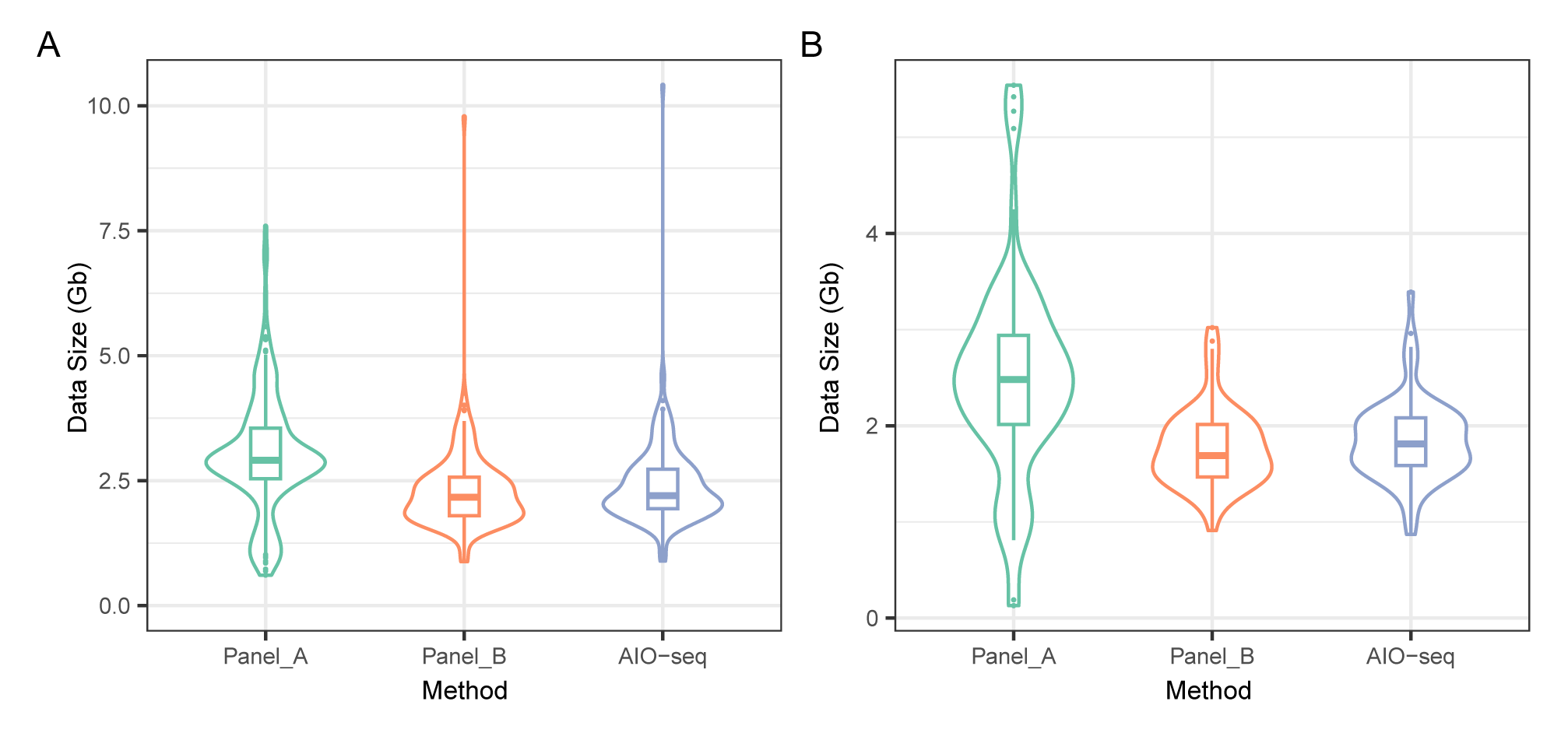

Supplement: Supplementary file 3 — Additional file 3: Figure S8. Sequencing yields of the maize genetic population.Sequencing yields of different sequencing strategies in the CIMBL83/GEMS41 population.Sequencing yields of different sequencing strategies in the CML496/GEMS41 population. For boxplots, center line is the median; box limits represent upper and lower quartiles. Different methods use different REs. Figure S9. iRAD significantly reduces the number of reads containing restriction enzyme recognition sites.Proportion of reads without AluI recognition sites in the CIMBL83/GEMS41 populationand CML496/GEMS41 populationwith Panel_A, Panel_B and AIO-seq.Proportion of reads without HindIII recognition sites in the CIMBL83/GEMS41 populationand CML496/GEMS41 populationwith Panel_A, Panel_B and AIO-seq. Different methods use different REs. Figure S10. iRAD-seq can enhance the depth of the obtained SNPs.The number of SNPs at different depths. The data used in the experiment was 0.5 Gb, 1.5 Gb, and 2.0 Gb. SNP number at different depths. Log10: n is the number of SNPs. For boxplots, center line is the median; box limits represent upper and lower quartiles. Employ the t-test to assess differences in different method, p < 0.0001. Different methods use different REs. [file 12915_2025_2330_MOESM3_ESM.zip › Fig S8.tif]

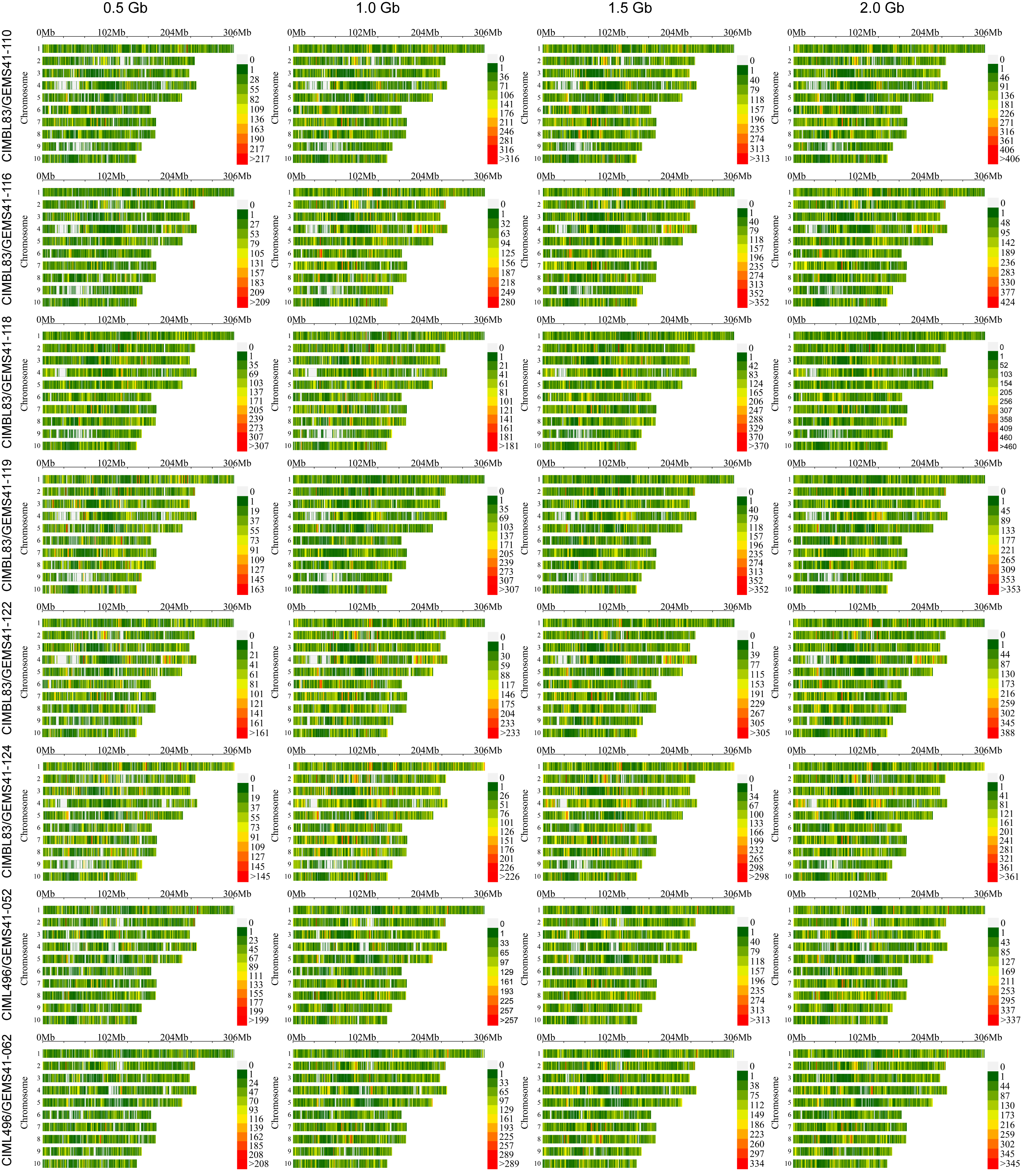

Supplement: Supplementary file 4 — Additional file 4: Figure S11. Chromosome distribution of SNPs when using varying sequencing data sizes with Panel_A. The size of the sliding window is 1 Mb. Red, high density; green, low density. [file 12915_2025_2330_MOESM4_ESM.zip › Fig S11.tif]

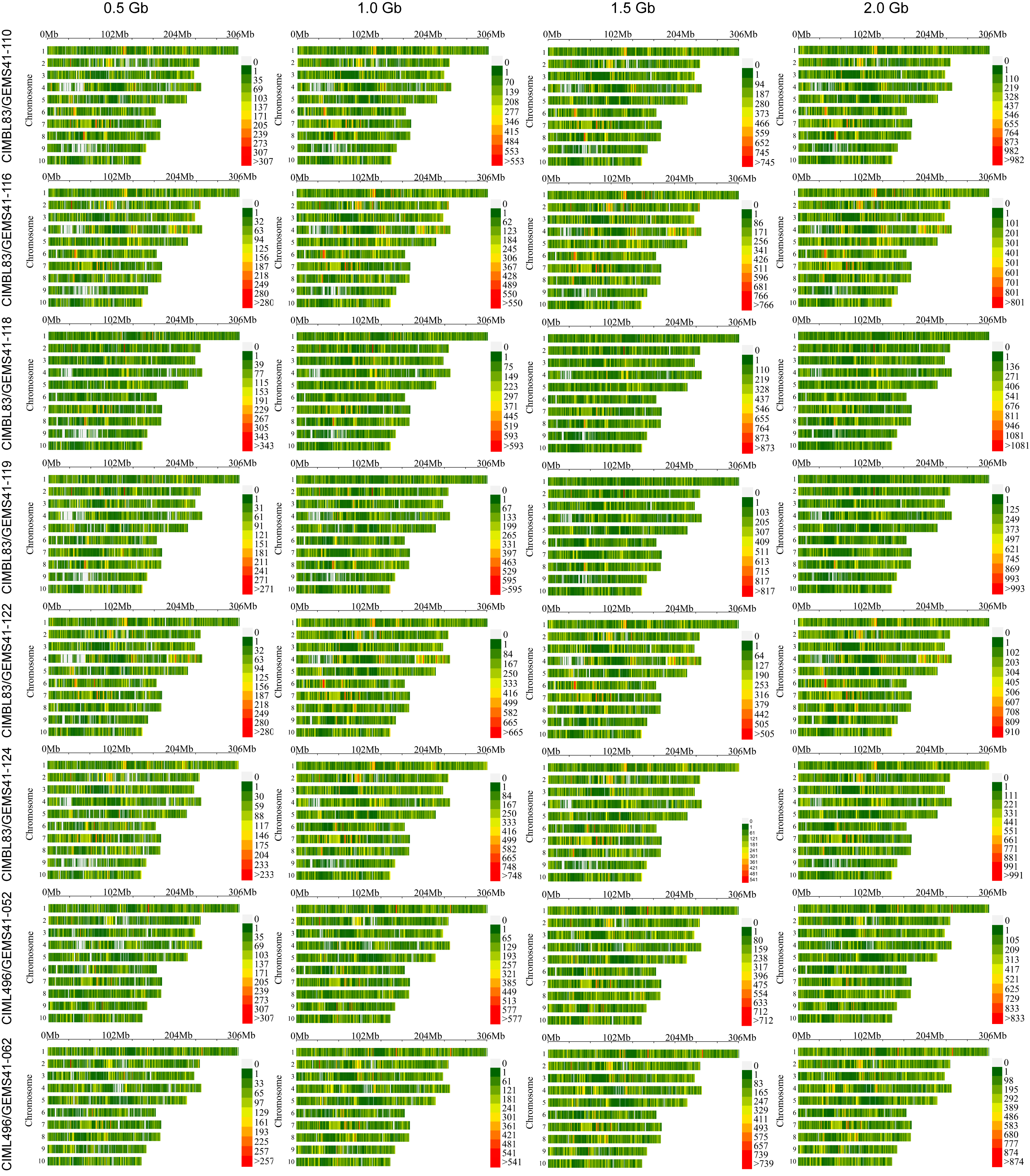

Supplement: Supplementary file 5 — Additional file 5: Figure S12. Chromosome distribution of SNPs when using varying sequencing data sizes with panel_B. The size of the sliding window is 1 Mb. Red, high density; green, low density. [file 12915_2025_2330_MOESM5_ESM.zip › Fig S12.tif]

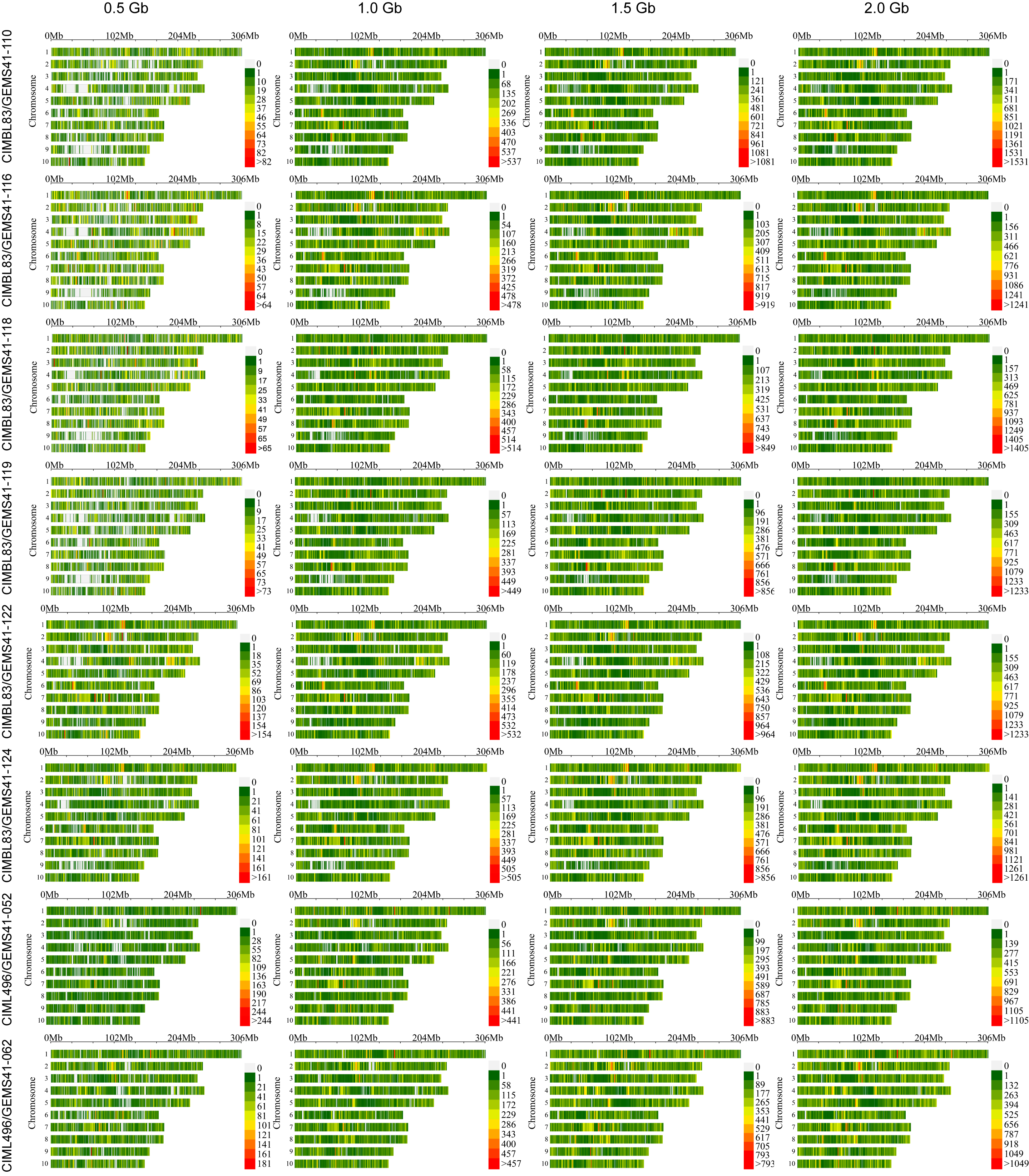

Supplement: Supplementary file 6 — Additional file 6: Figure S13. Chromosome distribution of SNPs when using varying sequencing data sizes with AIO-seq. The size of the sliding window is 1 Mb. Red, high density; green, low density. [file 12915_2025_2330_MOESM6_ESM.zip › Fig S13.tif]

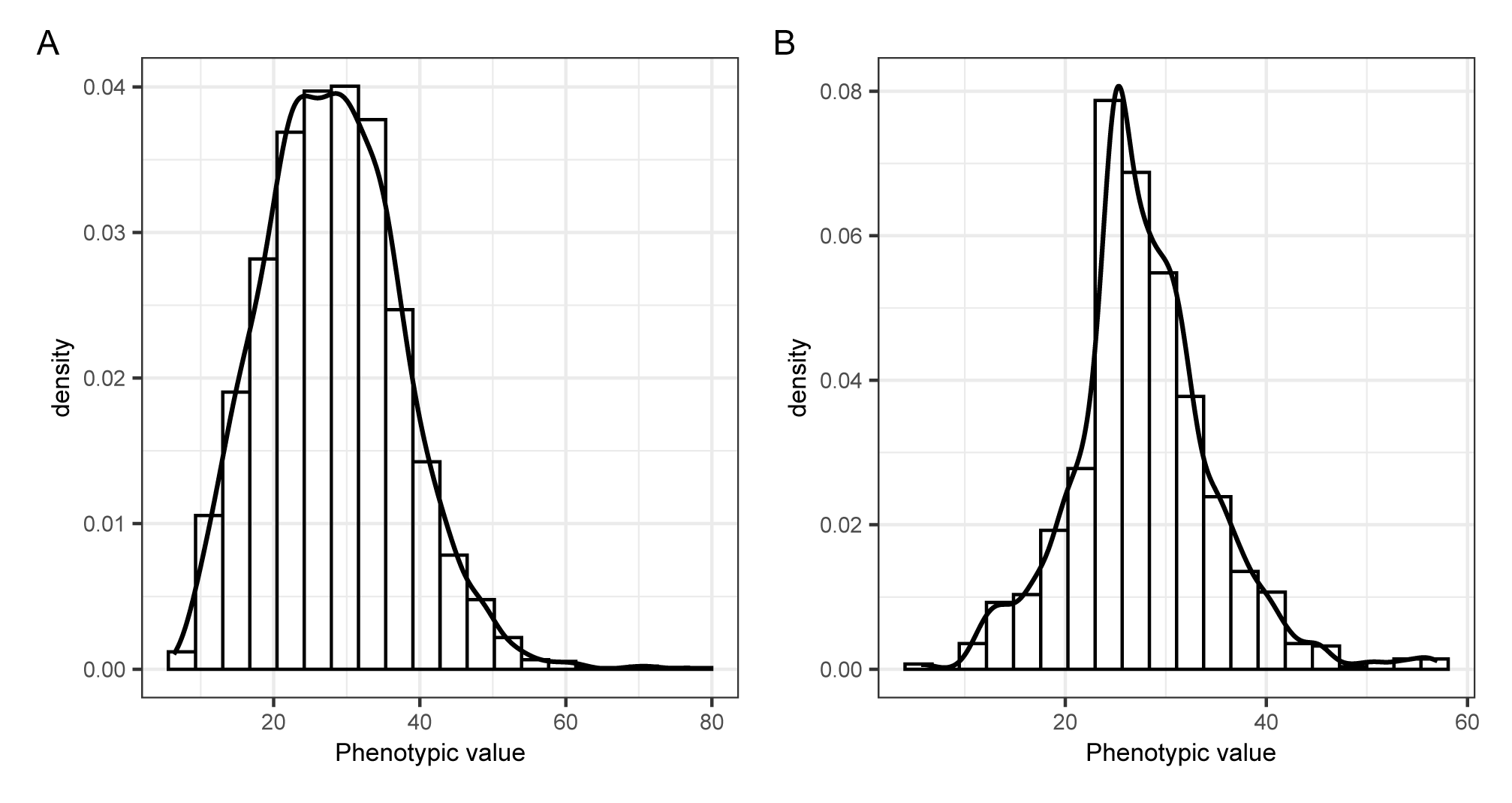

Supplement: Supplementary file 7 — Additional file 7: Figure S14. Bin maps and genetic maps of iRAD-seq.Construction of recombination bin maps illustrating recombination events in the CIMBL83/GEMS41and CML496/GEMS41 populationswith different sequencing strategies.High-density genetic maps of the CIMBL83/GEMS41and CML496/GEMS41 populationwith different sequencing strategies. Different methods use different REs. Figure S15. Phenotypic distribution of the leaf angle.Distribution of 193 maize lines from CIMBL83/GEMS41.The distribution of 68 maize lines from CML496/GEMS41. Figure S16. iRAD-seq workflow integrated with a liquid handling automation system. The estimated time required is based on the processing of 960 samples. [file 12915_2025_2330_MOESM7_ESM.zip › Fig S15.tif]

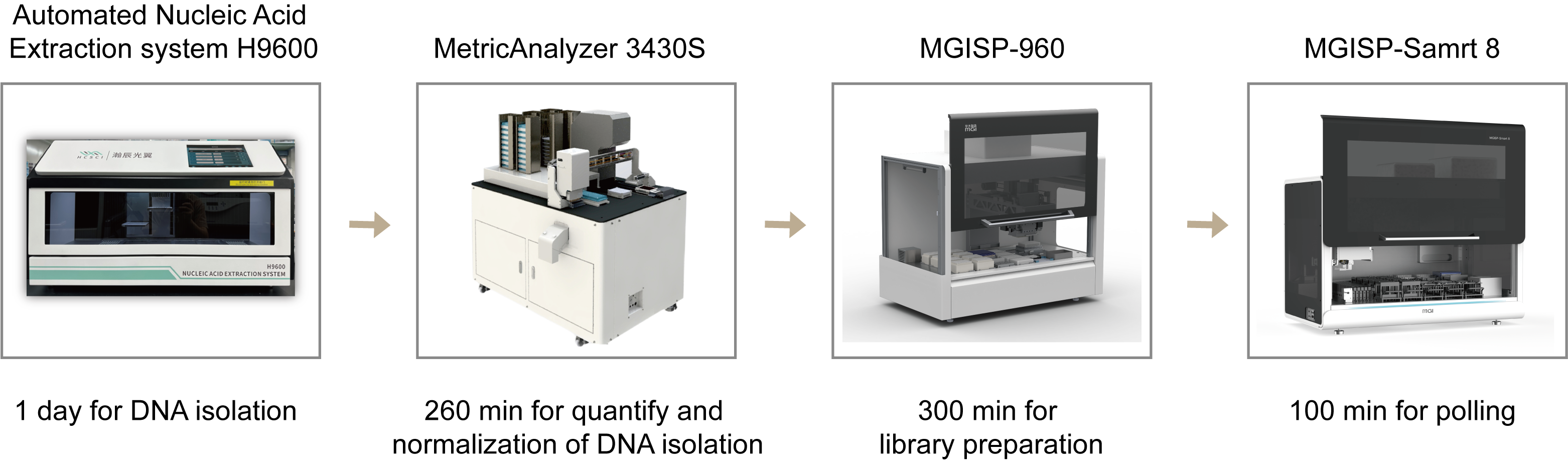

Supplement: Supplementary file 7 — Additional file 7: Figure S14. Bin maps and genetic maps of iRAD-seq.Construction of recombination bin maps illustrating recombination events in the CIMBL83/GEMS41and CML496/GEMS41 populationswith different sequencing strategies.High-density genetic maps of the CIMBL83/GEMS41and CML496/GEMS41 populationwith different sequencing strategies. Different methods use different REs. Figure S15. Phenotypic distribution of the leaf angle.Distribution of 193 maize lines from CIMBL83/GEMS41.The distribution of 68 maize lines from CML496/GEMS41. Figure S16. iRAD-seq workflow integrated with a liquid handling automation system. The estimated time required is based on the processing of 960 samples. [file 12915_2025_2330_MOESM7_ESM.zip › Fig S16.tif]

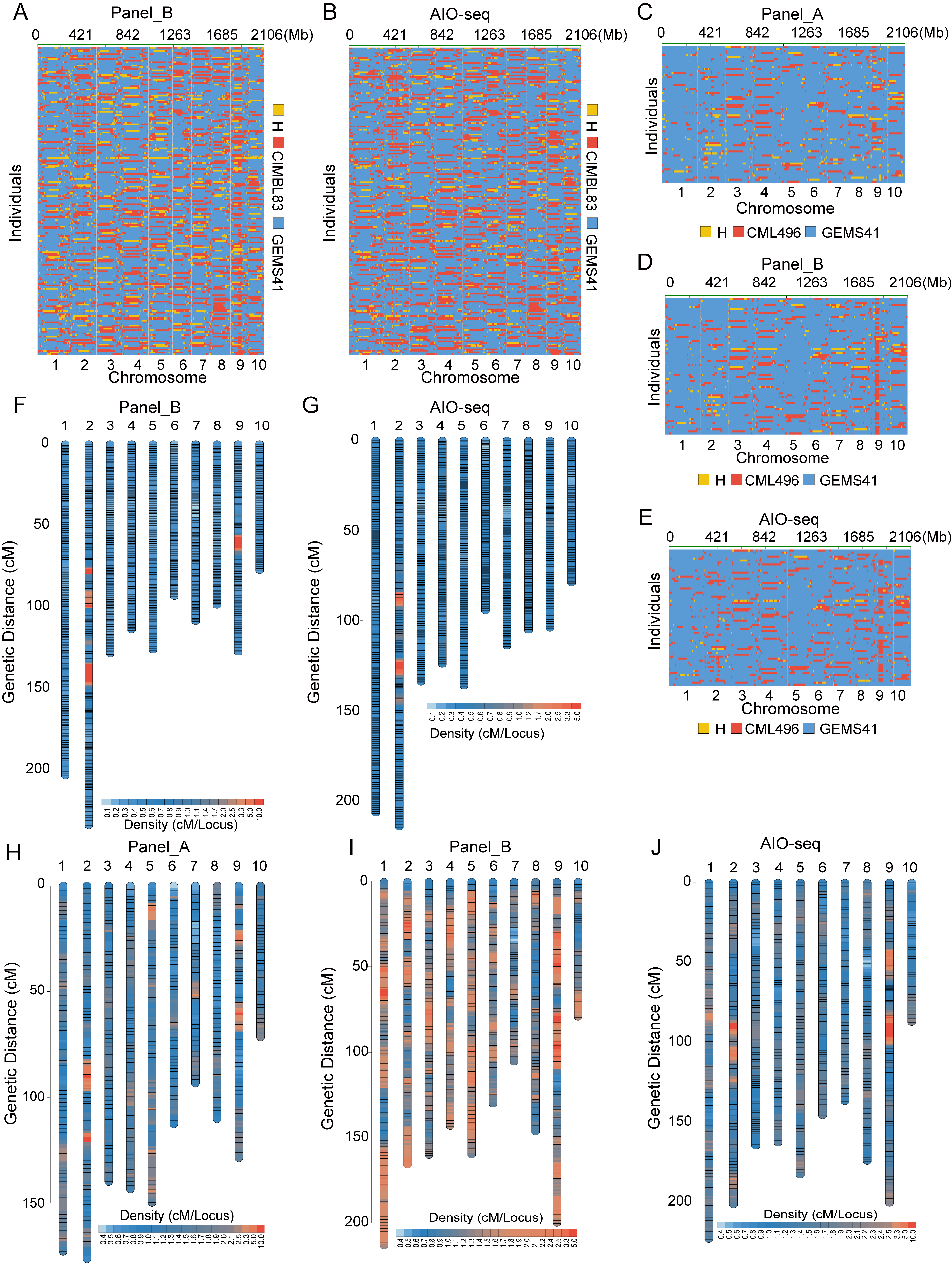

Supplement: Supplementary file 7 — Additional file 7: Figure S14. Bin maps and genetic maps of iRAD-seq.Construction of recombination bin maps illustrating recombination events in the CIMBL83/GEMS41and CML496/GEMS41 populationswith different sequencing strategies.High-density genetic maps of the CIMBL83/GEMS41and CML496/GEMS41 populationwith different sequencing strategies. Different methods use different REs. Figure S15. Phenotypic distribution of the leaf angle.Distribution of 193 maize lines from CIMBL83/GEMS41.The distribution of 68 maize lines from CML496/GEMS41. Figure S16. iRAD-seq workflow integrated with a liquid handling automation system. The estimated time required is based on the processing of 960 samples. [file 12915_2025_2330_MOESM7_ESM.zip › Fig S14.tif]
